# Insulin-like growth factor receptor signaling in tumorigenesis and drug resistance: a challenge for cancer therapy

**DOI:** 10.1186/s13045-020-00904-3

**Published:** 2020-06-03

**Authors:** Hui Hua, Qingbin Kong, Jie Yin, Jin Zhang, Yangfu Jiang

**Affiliations:** 1 State Key Laboratory of Biotherapy, Laboratory of Stem Cell Biology, National Clinical Research Center for Geriatrics, West China Hospital, Sichuan University, Chengdu, China; 2 State Key Laboratory of Biotherapy, Laboratory of Oncogene, Cancer Center, West China Hospital, Sichuan University, Chengdu, 610041 China

**Keywords:** Insulin-like growth factor, Cancer, Receptor tyrosine kinase, Tumorigenesis, Drug resistance

## Abstract

Insulin-like growth factors (IGFs) play important roles in mammalian growth, development, aging, and diseases. Aberrant IGFs signaling may lead to malignant transformation and tumor progression, thus providing the rationale for targeting IGF axis in cancer. However, clinical trials of the type I IGF receptor (IGF-IR)-targeted agents have been largely disappointing. Accumulating evidence demonstrates that the IGF axis not only promotes tumorigenesis, but also confers resistance to standard treatments. Furthermore, there are diverse pathways leading to the resistance to IGF-IR-targeted therapy. Recent studies characterizing the complex IGFs signaling in cancer have raised hope to refine the strategies for targeting the IGF axis. This review highlights the biological activities of IGF-IR signaling in cancer and the contribution of IGF-IR to cytotoxic, endocrine, and molecular targeted therapies resistance. Moreover, we update the diverse mechanisms underlying resistance to IGF-IR-targeted agents and discuss the strategies for future development of the IGF axis-targeted agents.

## Introduction

**Fig. 1:**
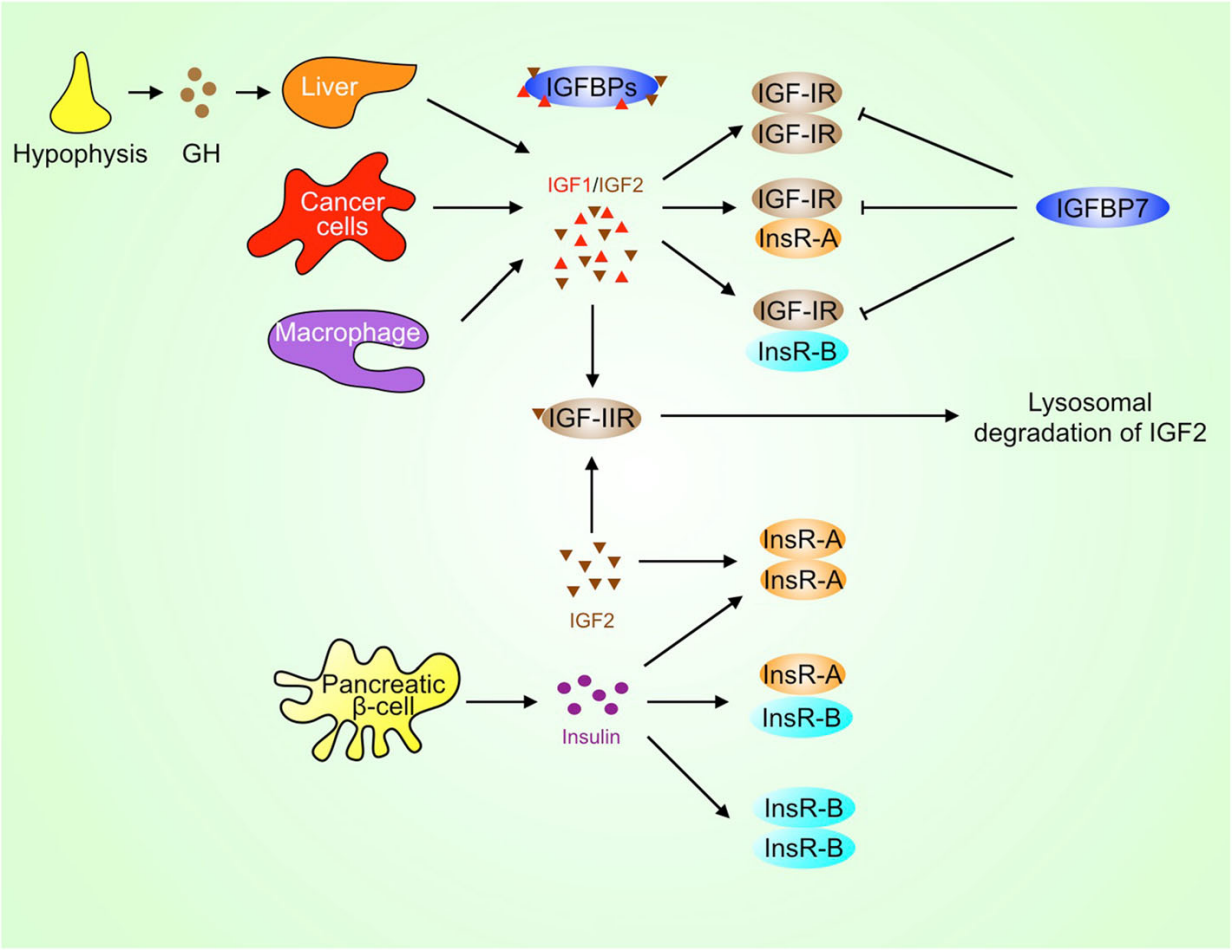
IGF, insulin, and their receptors. IGF can be secreted by the liver, cancer cells, and macrophages, while insulin is secreted by pancreatic β cells. IGF-IR may heterodimerize with InsR-A or InsR-B and then forms a hybrid receptor. Whereas IGFBPs usually act as serum reservoirs for IGFs, IGFBP7, in particular, may compete with IGFs to bind to IGF-IR and inhibit the activation of IGF-IR. GH, growth hormone

Sustained growth is a typical hallmark of cancer. Growth factors, such as epidermal growth factor (EGF), transforming growth factor (TGF), platelet-derived growth factor (PDGF), hepatocyte growth factor (HGF), fibroblast growth factor (FGF), vascular endothelial growth factor (VEGF), and insulin-like growth factor (IGF), stimulate cancer cell and stromal cell proliferation, migration, and invasion thereby promoting tumor growth, angiogenesis, and metastasis. While IGF deficiency may inhibit development and cause metabolic problems, excessive IGF levels disturb health. Increased IGF levels are inversely associated with longevity and positively associated with cancer risk. IGF is mainly produced by the liver, as well as tumor cells and cancer-associated macrophages (Fig. 1). Furthermore, the bioavailability of IGF is regulated by IGF-binding proteins (IGFBPs), which are identified as the serum reservoirs of IGF [1]. Epidemiological studies have demonstrated that circulating IGF and IGFBP levels are associated with some types of cancer. For example, higher levels of circulating IGF are associated with increased risk of breast and prostate cancer [2, 3]. Recent study also reveals that there is a modest positive association between IGF1 and lung cancer risk in current smokers [4]. Although IGF1 was not associated with overall colorectal cancer risk, it appears to be associated with the increased risk for advanced colorectal cancer [5]. In addition, serum levels of IGFBP3 are inversely associated with esophageal and gastrointestinal cancer [6, 7]. However, a recent nested case-control study reveals that circulating concentration of IGF1 is not associated with bladder cancer, lymphoma, and melanoma risk in a European population [8–10].

IGF binds to its cell surface receptors such as type I insulin-like growth factor receptor (IGF-IR) and then initiates multiple signaling pathways including PI3K/Akt, MAPK, JAK/STAT, Src, and focal adhesion kinase (FAK), which act in concert to stimulate cancer cell proliferation, survival and migration. Activation of IGF signaling pathways promotes the growth, metastasis, and drug resistance in many types of human tumors, including mesenchymal, epithelial, and hematopoietic cancer [11–13]. Previous study indicates that IGF-IR may be involved in cell-fate determination. Elevated expression of IGF-IR in BCR/ABL^+^ cells may promote the development and self-renewal of chronic myeloid leukemia, while downregulation of IGF-IR in BCR/ABL^+^ cells leads to acute lymphoblastic leukemia (ALL) [14]. However, another study demonstrates that IGF-IR overexpression is essential for leukemia-initiating cell activity in T-ALL [15]. In addition, Bcr-Abl can induce autocrine IGF1 signaling in leukemia cells [16]. Autocrine IGF1 signaling also promotes growth and survival of human acute myeloid leukemia cells [17]. Moreover, IGF-IR is involved in the progression of chronic lymphocytic leukemia [18]. IGF-IR interacts with NPM-ALK and then promotes T cell ALK^+^ anaplastic large cell lymphoma cells survival [19].

Given the important roles of IGF-IR in tumor progression, inhibition of IGF-IR activity has been proposed as a therapeutic strategy for solid tumors and hematologic malignancies [20]. Many anti-IGF-IR monoclonal antibodies (mAbs) and small-molecule inhibitors have been developed. Although anti-IGF-IR mAbs and IGF-IR inhibitors have potent anti-cancer effects in preclinical models, clinical trials of these agents are largely disappointing in unselected cancer patients. Nevertheless, some anti-IGF-IR mAbs, such as ganitumab, show clinical benefits in some types of cancer, and are still under active evaluation. In this review, we update recent progress in exploring the complex IGF signaling, its role in drug resistance, and the mechanisms for resistance to IGF-IR inhibition. We also revisit the strategies to target IGF-IR for cancer therapy.

## IGFs and their receptors

The physiological and pathological functions of IGF are largely mediated by its receptors. There are two types of IGF receptors, namely type I IGF receptor (IGF-IR) and type II IGF receptor (IGF-IIR). IGF-IR is a receptor tyrosine kinase (RTK) that mediates the stimulatory effects of IGF1 and IGF2 on cell proliferation, migration, and invasion. In contrast, IGF-IIR lacks the kinase activity. Therefore, IGF-IIR can sequester IGF2 and even deliver it to lysosome for degradation, thereby abrogating IGF2 signaling (Fig. 1) [21]. IGF-IR consists of extracellular α subunit (IGF-IRα) and transmembrane β subunit (IGF-IRβ). Both IGF-IRα and IGF-IRβ are encoded by a single gene on chromosome 15q26.3. After the translation of *IGF*-*IR* mRNA, the 210 kDa protein product (pro-IGF-IR) is subject to endoproteolytic cleavage by the pro-protein convertases such as furin and pro-protein convertase 5, leading to the generation of α chain (706 amino acids) and β chain (627 amino acids) [22]. The mature IGF-IR is a heterotetramer with two α chains and two β chains [23]. In addition, IGF-IR has high homology with the closely related insulin receptor (InsR), which has two isoforms, InsR-A and InsR-B [24]. The InsR-A is generated by alternative splicing of exon 11 in *InsR* gene [25]. InsR-A differs from InsR-B in ligand-binding and signaling properties. While InsR-A homodimer binds both insulin and IGF2 with high affinity, InsR-B homodimer and InsR-A/B heterodimer preferentially bind insulin but not IGF. IGF-IR may heterodimerize with InsR-A or InsR-B and then forms a hybrid receptor. IGF1/2 binds to IGF-IRα homodimer and IGF-IR/InsR heterodimer. Especially, IGFBP7 may compete with IGF to bind to the extracellular domain of IGF-IR and then suppress the activation of IGF-IR by IGF [26]. In addition, IGF2 can bind to InsR-A homodimer (Fig. 1) [27]. InsR-B, which contains 12 amino acids encoded by exon 11 of InsR, mediates the metabolic actions of insulin such as the uptake of glucose by muscle and adipose [25, 28]. To prevent metabolic disorders, targeting IGF-IR/InsR for cancer therapy should avoid compromising the function of InsR-B.

Upon binding to IGF, IGF-IR undergoes changes in its conformation, which in turn abolishes the restraints of intrinsic kinase activity by the ectodomain, and triggers transphosphorylation of its tyrosine kinase domains [29]. Phosphorylation of a triple-tyrosine cluster (Y1131/1135/1136) in the kinase domain of β subunit can further amplify the kinase activity of IGF-IR. Except for autophosphorylation, IGF-IR can be phosphorylated and activated by other kinases such as Src and FAK [30, 31]. Of note, the protein kinase mTOR has tyrosine kinase activity [32]. mTORC2 interacts with IGF-IR, and phosphorylates IGF-IR at Y1131/1136 thereby activating IGF-IR [32]. The conformational changes in IGF-IR create docking sites for its substrates, such as Shc and InsR substrates (IRS), which relay the signaling to downstream effectors including PI3K, MAPK, and STAT3. The IRS1/Akt pathway is critical for regulation of metabolism by insulin and InsR. Instead, InsR-A mediates the mitogenic effects of both IGF and insulin, which is dependent on receptor internalization, phosphorylation of SHC and MAPK [33].

IGF-IR has both beneficial and detrimental effects on health. Studies in IGF-IR-deficient mice demonstrate that these mice die within a few days after birth [34], indicating that IGF-IR has critical roles in development and health. However, overexpression of IGF-IR may induce cellular transformation. IGF-IR is frequently overexpressed or activated in a variety of cancer. IGF-IR expression is elevated in about 50% of breast cancers, and more frequently in luminal A-type breast cancer than luminal B and triple-negative breast cancer [35]. The prognostic impact of IGF-IR expression in human breast cancer remains inconclusive. While some studies suggest that overexpression of IGF-IR or phosphorylated IGF-IR is inversely associated with patient prognosis among all subtypes of breast cancer [36–38], another study reveals that IGF-IR is only inversely associated with prognosis in ErbB2-positive breast cancer [39]. One study even shows that luminal A/B breast cancer patients with high IGF-IRα and negative EGFR expression have better prognosis than the rest [40]. The reasons for discrepancy among these studies are unclear. It remains to know whether the levels of IGF-IRα and IGF-IRβ have different impact on the prognosis. One possibility is that the IGF-IRα/IGF-IRβ ratio can dictate the different outcome.

In addition, IGF-IR is overexpressed in about 30% of human prostate cancer. Overexpression of IGF-IR in prostate cancer is associated with high Gleason grade and increased risk of tumor recurrence and metastasis [41]. Moreover, cell membrane expression of IGF-IR is present in 36% of head and neck squamous carcinoma (HNSCC), while cytoplasmic IGF-IR is detected in 92% of HNSCC [42]. Regardless of the location of IGF-IR, high levels of IGF-IR are associated with high tumor stage, HPV negativity, and short overall survival [42]. Overexpression of IGF-IRβ was correlated with a decreased survival in patients with cervical carcinoma [43]. Except for the levels of IGF-IR, the IGF-activated gene transcription signature is strongly associated with poor prognosis in breast cancer patients [38]. Therefore, both the levels and activity of IGF-IR are positively correlated with tumor progression.

## The biologic effects of IGFs signaling on cancer

**Fig. 2:**
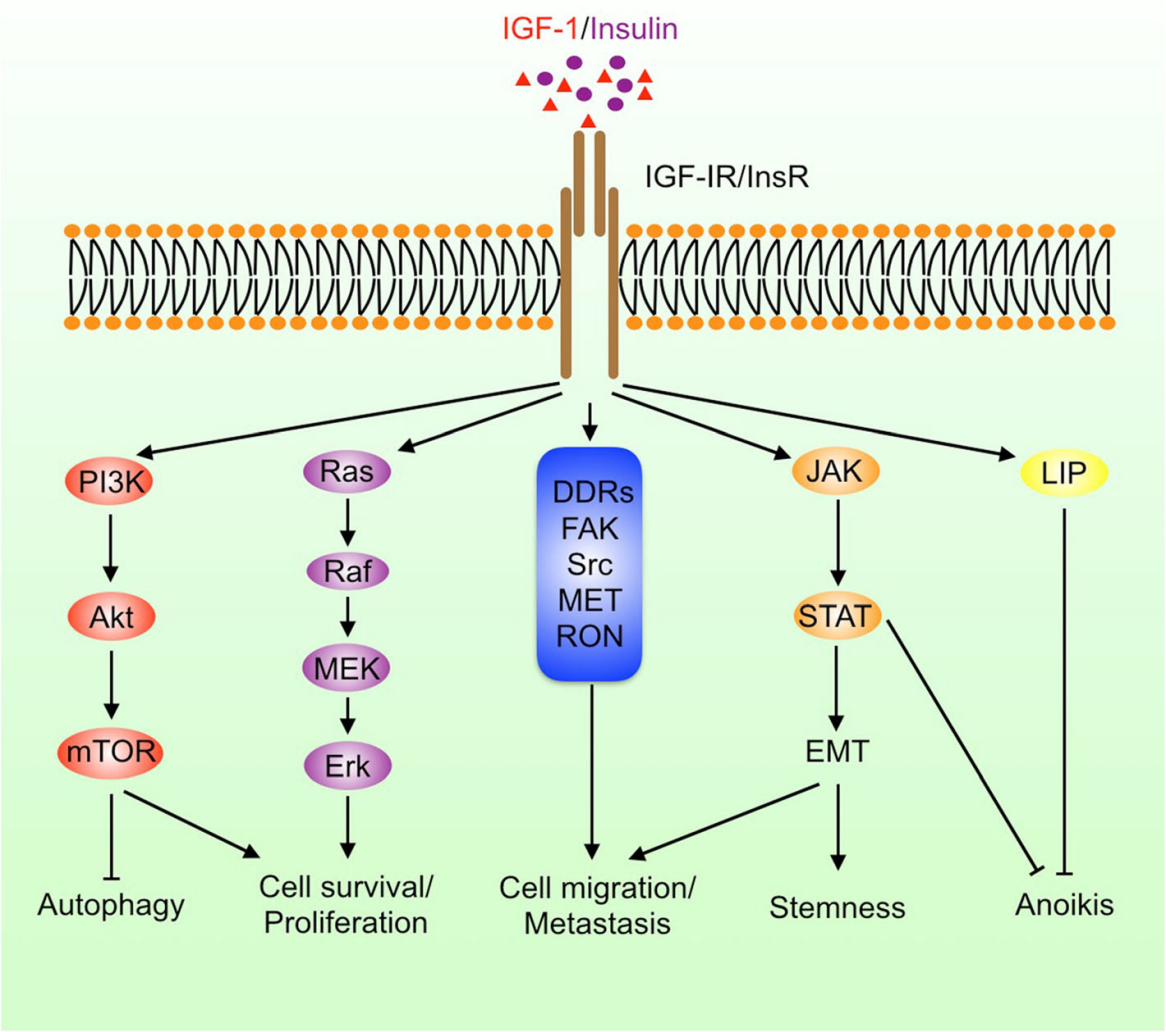
IGF and insulin signaling pathways. IGF and insulin can activate multiple signaling pathways including PI3K, MAPK, JAK/STAT, discoidin domain receptors (DDRs), FAK, and Src; promote cell proliferation, survival, epithelial-mesenchymal transition (EMT), migration, and stemness; and inhibit autophagy and anoikis

Once activated by the ligands, IGF-IR and InsR may initiate diverse signaling pathways to regulate cell proliferation, differentiation, survival, metabolism, migration, and stemness (Fig. 2). IRS1/2, Shc, and Grb, are typical adaptors to transduce signaling from IGF-IR to downstream effectors. While most of the canonical IGF-IR signaling processes are conducted in the cytosol, IGF-IR may translocate to the nuclei and then regulate gene transcription, which represents a non-canonical function of IGF-IR.

### IGF-IR promotes tumor growth, anoikis evasion, and metastasis

PI3K is a classical lipid kinase that catalyzes the production of phosphatidylinositol-3,4,5-trisphosphate (PtdIns-3,4,5-P_3_) [44]. Activated IGF-IR phosphorylates IRS1/2 at tyrosine residues and then promotes their association with PI3K, leading to increased PI3K activation, PIP3 abundance, and the activation of PDK1 by PIP3 [45]. In this case, IRS1/2 may act as protein scaffolds to lock PI3K in an active conformation. In addition, PI3K promotes mTORC2 activation [46]. While PDK1 phosphorylates Akt at Thr308, mTORC2 phosphorylates Akt at Ser473. Phosphorylation of Akt at both Thr308 and Ser473 leads to full activation of Akt, thereby regulating glucose metabolism, protein synthesis, cell proliferation, survival, and apoptosis through multiple targets [46]. After phosphorylation by Akt, Glut is translocated to cell membrane allowing glucose uptake. Besides, Akt phosphorylates and inactivates GSK3, FOXO, and TSC1/2, leading to activation of Wnt signaling, inhibition of apoptosis, and activation of mTORC1, respectively [47]. In addition, Akt phosphorylates Bcl2 to promote cell survival and inactivates p27 and Bad to inhibit cell cycle arrest and apoptosis.

Except for the PI3K pathway, the Ras-Raf-MEK-ERK pathway is also important for tumorigenesis. The adaptor Shc is an essential link between IGF-IR and Ras. IGF-IR induces tyrosine phosphorylation of Shc, which is assembled into a complex containing Grb2 and SOS. Subsequently, the Ras/Raf/MEK/ERK cascade is activated. ERK may promote cancer cell proliferation by activating other tumor-promoting proteins such as Yes-associated protein (YAP) [48]. In addition, the induction of YAP signaling by hypoxia is dependent on IGF-IR, which helps cancer cells adapt to hypoxia [49, 50].

Cancer cell detachment from the matrix is essential for metastasis. Upon detachment from the matrix, circulating tumor cells may be subject to anoikis. Upregulation of IGF-IR signaling can help cancer cells resist anoikis by inhibiting p53 and p21 activation [51]. IGF-IR also promotes anchorage-independent growth through RACK1-mediated STAT3 and Akt activation [52, 53]. Furthermore, IGF-IR/Akt signaling promotes the expression of LIP, an anoikis suppressive isoform of CCAAT enhancer binding protein-β [54]. Inhibition of IGF-IR enhances the susceptibility of cancer cells to anoikis, reduces circulating tumor cells in the blood, and inhibits cancer metastasis [55].

As a receptor tyrosine kinase, IGF-IR can activate a couple of tyrosine kinases that have important roles in tumor progression, such as Src, FAK, JAK, RON, and MET [56, 57]. FAK and JAK/STAT3 activation promote cancer metastasis. Indeed, overexpression and activation of IGF-IR is associated with high risk of metastasis and poor prognosis in many cancer patients [58]. Moreover, IGF-IR interacts with discoidin domain receptors, the non-integrin collagen receptors that promote EMT, cell proliferation, and survival [59, 60]. Both tumor growth and metastasis are dependent on angiogenesis. IGF promote angiogenesis through VEGF, an effector downstream of PKCδ and Akt that are activated by IGF-IR [61]. The tumor suppressor VHL can interact with PKCδ and prevent its binding to IGF-IR, thereby abrogating IGF-IR-PKCδ signaling [62]. Moreover, previous studies demonstrate that IGF promotes mitochondrial biogenesis and turnover in cancer cells by inducing peroxisome proliferator-activated receptor γ coactivator 1β (PGC-1β), PGC-1α-related coactivator, nuclear factor-erythroid-derived 2-like 2 (NFE2L2/NRF2), and the mitophagy mediator BNIP3 [63]. In addition, IGF induces the expression of the cystine/glutamate antiporter (SLC7A11), which promotes the uptake of cystine to counteract oxidation [64]. Thus, inhibition of IGF-IR may lead to impaired mitochondrial functions and increased the production of reactive oxygen species [65].

### IGF-IR promotes epithelial-mesenchymal transition (EMT) and stemness

It is conceived that dynamic EMT and mesenchymal-epithelial transition drive cancer cell invasion, survival in circulation, and outgrowth in secondary organs. EMT is characteristic of the downregulation of E-cadherin and upregulation of N-cadherin and vimentin. The transcription factors smad, zeb1/2, snail, slug, and twist are key drivers of the EMT program. Loss of the epithelial marker E-cadherin may impair cell-cell adhesion, cell-matrix adhesion, and cell polarity. IGF-IR is able to induce EMT through multiple mechanisms [66]. IGF-IR-activated Akt may stabilize slug, a negative regulator of E-cadherin expression. On the other hand, IGF-IR may trigger EMT via STAT3, FAK, and NF-kB [67, 68].

EMT may drive epithelial cells into a stem cell-like state [69]. Indeed, IGF-IR promotes both EMT and cancer stemness. Mechanistically, activation of STAT3 contributes to the induction of EMT and cancer stemness by IGF-IR [30, 70]. In addition, IGF-IR is overexpressed in cancer stem cells and stimulates the expression of stemness transcription factors such as inhibitor of DNA-binding 1, Nanog, and POU5F1 [71, 72]. Inhibition of the IGF-IR-Akt-mTOR pathway suppresses breast cancer stem/progenitors [73].

IGF-IR is also involved in the regulation of EMT and cancer stemness by oncogenes and tumor suppressor genes. DNp73, a dominant-negative variant of the tumor suppressor p73, enhances EMT and cancer stemness by abrogating the inhibitory effect of EPLIN on IGF-IR and downregulating miR-885-5p thereby increasing its direct target IGF-IR [74, 75]. In addition, NF-kB can induce IGF2 expression in cancer cells to activate IGF-IR, and then promote cancer stemness. During stroma-cancer cells crosstalk, cancer-associated fibroblasts may secrete IGF2 to activate IGF-IR/Nanog axis in tumor cells and promote cancer stemness [76]. Lastly, fibulin 3 (FBLN3), an extracellular matrix protein, suppresses both EMT and self-renewal of the lung cancer stem cells by inhibiting the IGF-IR/PI3K/AKT/GSK3β pathway [77].

### Nuclear IGF-IR regulates gene expression

While IGF-IR is usually localized at the plasma membrane in normal cells, it often translocates into the cytoplasm and nucleus in cancer cells even though it does not have the nuclear localization signal [78–80]. Although the SUMOylation of IGF-IR at three evolutionarily conserved lysine residues (K1025/1100/1120) in IGF-IRβ is dispensible for its kinase activity, SUMOylation is essential for its nuclear translocation [81]. In addition, the nuclear translocation of IGF-IR is dependent on the SUMO-conjugating enzyme Ubc9 [82]. Mechanistically, the dynactin subunit p150(Glued) transports IGF-IR to the nuclear pore complex, where importin-β and RanBP2 interact with IGF-IR and then deliver it into the nucleus [83]. Moreover, the growth factor amphiregulin interacts with IGF-IR and promotes the binding of IGF-IR to importin-β1 thereby enhancing the nuclear translocation of IGF-IR [84]. Nuclear IGF-IR can not only bind to IGF-IR promoter DNA and promotes the transcription of itself [85], but also directly binds DNA and recruit RNA polymerase II to upregulate JUN and FAM21 expression in cancer cells [86]. In addition, IGF-IR interacts with T cell factor (TCF) and promotes the expression of β-catenin/TCF targets including cyclin D1 and axin 2 [87]. Of note, the kinase activity of IGF-IR is not required for its nuclear translocation and activation of transcription [88]. Nevertheless, the nuclear IGF-IR still acts as a kinase to phosphorylate its nuclear partners. For example, nuclear IGF-IR phosphorylates proliferating cell nuclear antigen at Y60/133/250 and promotes its ubiquitination, which prevents replication fork stalling after DNA damage [89]. Moreover, nuclear IGF-IR promotes slug expression through phosphorylating histone H3 at Y41 [90].Thus, nuclear IGF-IR has both kinase-dependent and kinase-independent functions.

## IGF-IR promotes drug resistance in cancer therapy

IGF-IR not only promotes cell proliferation, but also prevents stress-induced cell death. Previous studies demonstrate that IGF-IR contributes to resistance to various category of cancer therapeutics including chemotherapy, endocrine therapy, radiotherapy and targeted therapy. Overexpression and activation of IGF-IR during chemotherapy, radiotherapy or targeted therapy predicts poor outcome in cancer patients [91]. Meanwhile, preclinical studies indicate that inhibition of IGF-IR may reverse the resistance to many drugs and improve the efficacy of those anti-cancer agents.

### Radiotherapy and chemotherapy

DNA damage cancer therapy is one of classical cancer treatments. IGF-IR is able to repair DNA damage through both non-homologous end joining (NHEJ) and homologous recombination [92]. Inhibition of IGF-IR impairs the repair of radiation-induced double strand breaks and sensitizes cancer cells to radiotherapy [82]. The promotion of NHEJ by IGF-IR may rely on DNA-dependent protein kinase (DNA-PK), whereas a direct interaction between IGF-IR and DNA-PK is absent [93]. Of note, the IGF-IR downstream effectors Akt and mTOR promote the repair of radiation-induced DNA double strand breaks and confer resistance to radiotherapy [93, 94]. Inhibition of residual homologous recombination further enhances the sensitivity to IGF-IR inhibitors [95]. Since IGF-IR can promote DNA damage repair, activation of IGF-IR may contribute to resistance to DNA-damaging agents. Hence, inhibition of IGF-IR represents a therapeutic strategy to target DNA repair and sensitize cancer cells to DNA-damaging agents. Indeed, IGF-IR inhibition sensitizes cancer cells to ATM-related kinase inhibition, cisplatin, oxaliplatin, and doxorubicin [96–99]. Combined treatment with IGF-IR inhibitor, PARP inhibitor, and/or platinum may be a strategy to improve the efficacy of cancer therapy [100]. In addition, IGF-IR inhibition sensitizes cancer cells to other chemotherapeutic agents, such as paclitaxel, 5-fluorouracil (5FU), temozolomide, gemcitabine, and bortezomib [101–105]. Combination of IGF-IR inhibitor and these chemotherapeutic agents may result in increased DNA double strand breaks, apoptosis, and mitotic catastrophe. Except for repression of DNA repair, IGF-IR inhibition may downregulate the synthesis of survivin, a dual regulator of cell proliferation and survival [106]. Decreased expression of survivin contributes to the sensitization of cancer cells to chemotherapy by IGF-IR inhibitors.

### Endocrine therapy

In addition to neoadjuvant chemotherapy, endocrine therapy has important roles in treating some types of cancer such as breast cancer and prostate cancer. However, estrogen receptor α-positive (ERα+) breast cancers and androgen receptor-positive prostate cancer may adapt to hormone deprivation and acquire resistance to antiestrogen or antiandrogen therapies. Phosphorylation of IGF-IR/InsR is elevated in ERα+ breast cancer cells resistant to estrogen deprivation and tamoxifen, possibly due to increased secretion of IGF1/2 in these cells and elevated expression of retinoblastoma-binding protein 2 that inhibits IGFBP4/5 by interacting with ER-NRIP1-HDAC1 complex [107–109]. In addition, the dual InsR/IGF-IR inhibitor linsitinib can prevent the emergence of breast cancer cells that are resistant to long-term estrogen deprivation or antiestrogen treatment and inhibit growth of established ER+ breast cancer xenografts in ovariectomized mice [107]. Combination of linsitinib and the ER antagonist fulvestrant is more effective to inhibit hormone-independent breast cancer growth than the single agent [108]. Of note, treatment with the neutralizing IGF-IR monoclonal antibody is incapable of overcoming resistance to hormone deprivation or antiestrogen therapy, due to the compensation by InsR [107]. Given that ErbB contributes to IGF-IR/InsR and ER antagonist resistance, co-targeting ErbB, IGF-IR/InsR, and ER may achieve maximal anti-cancer effect on ER+ breast cancer [110].

Androgens and the androgen receptor have critical roles in prostate cancer development and progression. Androgen deprivation therapy is a conventional treatment for advanced prostate cancer. However, most prostate cancers eventually become resistant to androgen deprivation and progress to castration-resistant disease. IGF2 mRNA expression is increased in prostate cancer during progression to castration-resistant disease [111]. Moreover, androgen synthesis is upregulated by IGF2 that increases the expression of steroidogenic enzymes including steroid acute regulatory protein, cytochrome p450 family member 17A1, aldo-keto reductase family member 1C3, and hydroxysteroid dehydrogenase 17B3 [111, 112]. The intratumoral androgen synthesis may help prostate cancer adapt to castration and progress to castration resistance [113]. Combination of the anti-IGF-IR Ab ganitumab and castration potently inhibits prostate cancer in animal model [113]. In addition, the androgen synthesis inhibitor abiraterone is a treatment for metastatic castration-resistant prostate cancer. IGF-IR phosphorylation is elevated in prostate cancer cells and residual resistant tumors after treatment with abiraterone [114]. Accordingly, IGF-IR inhibition can enhance the efficacy of castration and abiraterone on prostate cancer [114, 115].

### Molecularly targeted therapy

As a receptor tyrosine kinase, IGF-IR cross-talks with other RTKs such as EGFR, ErbB2, ErbB3, and MET. These RTKs share some common nodes among the signal transduction pathways. Inhibiting one of these RTKs may trigger system rewiring that allows other RTKs to compensate it. While EGFR family members can compensate IGF-IR inhibition and confer resistance to IGF-IR-targeted agents, IGF-IR can confer resistance to EGFR/ErbB2/ErbB3-targeted therapies as well. For example, IGF-IR and ErbB3 were significantly upregulated in ovarian cancer cells resistant to the anti-ErbB2 mAb trastuzumab [116]. Upon treating mucinous lung adenocarcinoma with the EGFR inhibitor gefitinib, the EGFR ligand amphiregulin promotes the binding of IGF-IR to importin-β1 and triggers nuclear localization of IGF-IR, which in turn prevents the induction of apoptosis by gefitinib [117]. Concomitant inhibition of both EGFR and InsR/IGF-IR is required to induce apoptosis in glioblastoma [118]. In addition, cholangiocarcinoma cells can adapt to EGFR inhibition by upregulation of IGF2-IR/IGF-IR axis [119]. Moreover, compensatory activation of IGF-IR is involved in resistance to co-inhibition of EGFR and MEK in KRAS-mutated colorectal cancer cells [120]. The IGF2/IGF-IR signaling confers resistance to anti-VEGF therapy, which can be circumvented by the dual InsR/IGF-IR inhibitor BI885578 [121]. Lastly, IGF signaling contributes to resistance to the multikinase inhibitor sorafenib in hepatocellular carcinoma [122].

PI3K/Akt/mTOR pathway is attractive target for cancer therapy. However, the clinical responses to PI3K inhibitors monotherapy are not as efficient as expected. IGF1 and IGF2 are overexpressed in PIK3CA-mutant breast cancer, which may activate IGF-IR signaling [123]. On the other hand, PI3K inhibition may abrogate the suppression of GSK3β and FOXO by Akt thereby promoting IGF-IR expression [124, 125]. IGF-IR inhibitor sensitizes PIK3CA-mutant breast cancer to PI3K inhibitors [123]. Moreover, IGF-IR phosphorylation is elevated in breast cancer cells resistant to the p110α isoform-selective inhibitor BYL719, making the BYL719-resistant cells sensitive to IGF-IR inhibition [126]. Upregulation of IGF-IR is detected in ovarian cancer cell that acquire resistance to the p110β isoform-selective inhibitor taselisib and in PI3K-δ inhibitor idelalisib-resistant chronic lymphocytic leukemia [125, 127]. Inhibition of IGF-IR overcomes resistance to taselisib and idelalisib in ovarian cancer and chronic lymphocytic leukemia [125–127]. Besides, IGF-IR confers resistance to mTOR inhibitors [128, 129]. Concurrent inhibition of both IGF-IR and mTOR more effectively suppresses tumorigenesis.

The resistance to MAPK, CDK, and ALK inhibitors also involves IGF-IR. BRAF, MEK, and Erk are important targets for treating BRAF-mutant melanoma and other cancers. Upregulation of IGF-IR is detected in melanoma that acquires resistance to the Erk1/2 inhibitor SCH772984, the BRAF inhibitor vemurafenib, and the MEK inhibitor trametinib [130]. Stimulation of the MEK5/Erk5 axis by IGF-IR bypasses the inhibition of BRAF, MEK1/2, and Erk1/2 [130]. In addition, upregulation of IGFBP2 expression may promote IGF-IR/Akt activation thereby conferring resistance to BRAF and MEK inhibitors [131]. Combination of IGF-IR and RAF/MEK/Erk inhibitors may improve the efficacy in cancer therapy. Moreover, IGF-IR activation is a mechanism underpinning the resistance to CDK4/6 inhibitor in Ewing sarcoma [132]. Combined treatment with CDK4/6 and IGF-IR inhibitors synergistically inhibits Ewing sarcoma [132]. Besides, selective anaplastic lymphoma kinase (ALK) inhibitors is a first-line therapy for nucleophosmin (NPM)-ALK-positive T cell lymphoma and echinoderm microtubule-associated protein like 4 (EML4)-ALK-positive non-small cell lung cancer (NSCLC) [133, 134]. IGF-IR physically interacts with NPM-ALK and enhances its phosphorylation/activation [19, 134]. Increased expression or activation of IGF-IR is detected in some cancer cell lines resistant to ALK inhibitors [133, 135]. Concurrent inhibition of both IGF-IR and ALK can combat ALK inhibitors resistance [133, 135].

Epigenetic dysregulation has emerged as one of the transcriptional vulnerabilities in human cancer. As coactivators that connect acetylated transcription factors and histones to the activation of RNA polymerase II, the bromodomain and extra-terminal domain (BET) family proteins preferentially promotes the transcription of oncogenes such as Myc, HER3, MET, and IGF-IR [136–138]. In addition, BET proteins enhance EWS-FLI1 activities in Ewing sarcoma. Therefore, BET inhibitors hold promise in treating cancer. However, activation of IGF-IR/Akt pathway compromises the efficacy of BET inhibitors in Ewing sarcoma. Combined treatment with BET and IGF-IR pathway inhibitors achieves potent and durable response in preclinical models [139]. Furthermore, the enhancer of zeste homolog (EZH2) is a histone methyltransferase that function as a transcriptional repressor. EZH2 mutation or overexpression is detected in solid tumors and leukemia, and correlated with cancer metastasis [140–142]. Hence, EZH2 inhibitors are emerging anti-cancer agents. However, activation of IGF-IR may confer resistance to EZH2 inhibitors in diffuse large B cell lymphoma [143].

## Clinical testing of IGF-IR-targeted agents

The identification of IGF-IR as an important cancer-promoting protein has stimulated the development of many IGF-IR-targeted agents, including anti-IGF-IR mAbs and small-molecule inhibitors. Given the promising anti-cancer effects of IGF-IR-targeted agents in preclinical models, clinical trials have been initiated to evaluate the efficacy of anti-IGF-IR mAbs (Table 1) [144–151]. However, anti-IGF-IR mAbs monotherapy is largely ineffective in unselected cancer patients. Some clinical trials suggest that IGF-IR inhibition may be effective only in selective cancer patients [152, 153]. A meta-analysis of 311 Ewing sarcoma patients in multiple trials demonstrates that treatment with anti-IGF-IR mAb figitumumab, R1507, or ganitumab results in complete response in 0.7% cases, partial response in 11% cases, and stable diseases in 21% cases [154]**.** Clinical evaluation of ganitumab remains active in patients with Ewing sarcoma, rhabdomyosarcoma and breast cancer. In fact, ganitumab has been approved as orphan drug in Ewing sarcoma by the US Food and Drug Agency. It is being tested to combine ganitumab with Src inhibitor dasatinib in patients with embryonal and alveolar rhabdomyosarcoma, and with chemotherapy, radiotherapy or CDK4/6 inhibitor in Ewing sarcoma (Table 1). In addition, combination of ganitumab with metformin is being tested in an ongoing I-SPY2 trial, which aims to identify  most effective agents to treat various types of breast cancer and learn about the predictors of treatment response [155]. Moreover, a randomized clinical trial in patients with advanced pancreatic carcinoma demonstrates that combination of the anti-IGF-IR mAb MK-0646 with gemcitabine is tolerable and associated with improvement in overall survival but not progression-free survival as compared with gemcitabine plus erlotinib [151]. As an alternative strategy to target IGF signaling, results of phase 1 clinical trials of the dual IGF1/2-neutralizing antibodies have been reported, while no results of phase 2/3 trials in cancer therapy have been reported [156, 157]. Currently, the dual IGF1/2-neutralizing antibody xentuzumab in combination with everolimus and exemestane is subject to clinical trial in ER^+^/ErbB2^-^ breast cancer. Combination of everolimus with exemestrane is an effective treatment for ER^+^/ErbB2^-^ breast cancer. It remains to know whether xentuzumab may further improve the efficacy of everolimus and exemestrane.

**Table 1:** Clinical evaluation of anti-IGF-IR or anti-IGF1/2 mAbs in cancer patients

Drug	Combination	Cancer type	Phase	Participants	Response	Ref. or trial ID
Ganitumab	None	Ewing sarcoma; desmoplastic small round cell tumors	2	38	PR, 16%; SD > 24 weeks: 49%.	[144] NCT00563680
Ganitumab	Doxorubicin, etoposide or radiotherapy	Metastatic Ewing sarcoma	3	330	This trial is still ongoing.	NCT02306161
Ganitumab	Palbciclib (CDK4/6 inhibitor)	Ewing sarcoma	2	18	This trial is still ongoing.	NCT04129151
Ganitumab	Dasatinib	Rhabdomyosarcoma	1/2	40	This trial is still ongoing.	NCT03041701
Ganitumab	Panitumumab	Colorectal cancer	1/2	142	ORR, 22%. No improvement, compared to panitumumab plus placebo.	[145] NCT00788957
Ganitumab	None	Recurrent platinum-sensitive ovarian cancer	2	61	Objective response rate, 1.6% (95%CI 0-8.8%); CBR, 1.7% (95% CI, 0-8.9%); PFS, 1.94 months (95% CI 1.45-2.1 months).	NCT00719212
Ganitumab	Gemcitabine	Metastatic pancreatic cancer	3	640	No improvement on OS.	[146] NCT01231347
Ganitumab	Metformin	Breast cancer	2	Estimated enrollment of 4000	This trial is still ongoing.	NCT01042379
Figitumumab	None	Ewing sarcoma	2	107	PR, 14.02%; SD, 23.36%.	[147] NCT00560235
Figitumumab	None	Ewing sarcoma	1	16	CR, 6.25%; PR, 6.25%; SD, 37.5%.	[148] NCT00474760
Cixutumumab	None	Refractory solid tumors	2	36	PR, 8.33%; SD, 13.89%.	[149]
Cixutumumab	None	Previously treated advanced or metastatic rhabdomyosarcoma, leiomyosarcoma, adipocytic sarcoma, synovial sarcoma or Ewing family of tumors	2	113	12-week PFR, 12% for rhabdomyosarcoma, 14% for leiomyosarcoma, 32% for adipocytic sarcoma, 18% for synovial sarcoma and 11% for Ewing family of tumors. Median PFS, 6.1 weeks for rhabdomyosarcoma, 6.0 weeks for leiomyosarcoma, 12.1 weeks for adipocytic sarcoma, 6.4 weeks for synovial sarcoma and 6.4 weeks for Ewing family of tumors.	[150]
Cixutumumab	None	Metastatic melanoma of the eye	2	18	CR, 0; PR, 0; SD, 50%; PFS, 2.21 weeks (95% CI 0–23.2); OS, 59.71 weeks (95% CI 0–109.6)	NCT01413191
Cixutumumab	Temsirolimus	Bone and soft tissue sarcoma	2	159	PR, 2.52%; SD, 61.64%; PD,35.85%	NCT01016015
MK-0646	Gemcitabine, Erlotinib	Advanced pancreatic carcinoma	1/2	45	PFS, 1.8 months (95% CI 1.8–9.7) for MK plus gemcitabine (arm A), 1.8 months (95% CI 1.7–5.5) for MK plus gemcitabine and erlotinib (arm B), and 1.9 months (95% CI 1.8–5.4) for gemcitabine and erlotinib (arm C); OS, 10.4 months (95% CI 3.9–18.9) for arm A, 7.1 months (95% CI 5.2–20.0) for arm B, and 5.7 months (95% CI 4.0–9.5) for arm C.	[151] NCT00769483
Xentuzumab	Everolimus, exemestane	Breast cancer	2	100	This trial is still ongoing.	NCT03659136
Xentuzumab	Everolimus, exemestane	Breast cancer	1b/2	164	This trial is still active.	NCT02123823
Xentuzumab	None	Advanced solid tumors	1	21	This trial is still active.	NCT02145741

*CR* complete response, *CBR* clinical benefit rate, *ORR* overall response rate, *OS* overall survival, *PFR* progression-free survival rate. *PFS* progression-free survival, *PR* partial response, *SD* stable disease, *Trial ID* registered number at ClinicalTrials.gov

Except for anti-IGF-IR or anti-IGF1/2 mAbs, many small-molecule IGF-IR inhibitors have been developed [158–163]. The most extensively evaluated IGF-IR inhibitor is linsitinib (OSI-906), a dual IGF-IR/InsR inhibitor (Table 2). A phase 2 trial in recurrent small cell lung cancer patients shows that linsitinib has no clinical activity in these patients, although it is a safe and tolerable treatment [159]. The response to linsitinib is inferior to the topoisomerase inhibitor topotecan, with median progression-free survival of 3.0 (95% CI 1.5–3.6) and 1.2 (95% CI 1.1–1.4) months for topotecan and linsitinib, respectively [159]. Besides, linsitinib fails to show activity in patients with metastatic prostate cancer, locally advanced or metastatic adrenocortical carcinoma [158, 161]. Combination of linsitinib with everolimus shows no objective response in patients with refractory metastatic colorectal cancer [160]. In addition, combination of linsitinib with erlotinib did not improve the progression-free survival and overall survival in patients with advanced non-small cell lung cancer, compared to erlotinib alone (Table 2). According to the recently reported results of a phase 2 trial in patients with gastrointestinal stromal tumors, linsitinib achieved no objective responses in these patients, although the rate of 9-month clinical benefit, progression-free survival, and overall survival at 9 months was 40%, 52% and 80%, respectively [162]. Despite these disappointing results, an early phase study suggests that AXL1717 may produce prolonged stable disease and survival of patients with relapsed malignant astrocytomas [163].

**Table 2:** Clinical evaluation of small-molecule IGF-IR inhibitors in cancer patients

Drug	Combination	Cancer type	Phase	Participants	Response	Ref. or trial ID
Linsitinib (OSI-906)	None	Locally advanced or metastatic adrenocortical carcinoma	3	139	OS, 323 days (95% CI 256–507) for linsitinib, 356 days (95% CI 249–556) for placebo	[158] NCT00924989
Linsitinib	None	Recurrent small cell lung cancer	2	44	SD, 3.45%; PFS, 1.2 months (95% CI 1.1–1.4); OS, 3.4 months (95% CI 1.8–5.6). The response to linsitinib is inferior to topotecan.	[159] NCT01533181
Linsitinib	Everolimus	Refractory metastatic colorectal cancer	1	18	No objective responses to treatment. PFS, 8 weeks (95% CI 7–9); OS, 30.6 weeks (95% CI 16.7–32.1)	[160] NCT01154335
Linsitinib	None	Metastatic prostate cancer	2	17	PSA partial response, 5.88%; PR, 10%; SD,80%; PD, 10%; PFS, 4.7 months (95% CI 3–6.7)	[161] NCT01533246
Linsitinib	Erlotinib	Advanced non-small cell lung cancer	2	88	Combination of linsitinib with erlotinib resulted in an increase in the incidence of renal and hepatic toxicities compared to erlotinib alone. Combination of linsitinib with erlotinib did not improve the PFS and OS compared to erlotinib alone.	NCT01221077
Linsitinib	None	Gastrointestinal stromal tumors	2	20	CR + PR + SD (≥ 9 months), 40%; PFS rate at 9 months, 52%; OS rate at 9 months, 80%	[162] NCT01560260
AXL1717	None	Recurrent or progressive malignant astrocytoma	1	9	Tumor response rate, 44%; SD for 12 months, 22.2%	[163] NCT00562380

*CR* complete response; *OS* overall survival, *PD* progressive disease, *PFS* progression-free survival, *PR* partial response, *SD* stable disease, *Trial ID* registered number at ClinicalTrials.gov

## IGF-IR inhibitors resistance

**Fig. 3:**
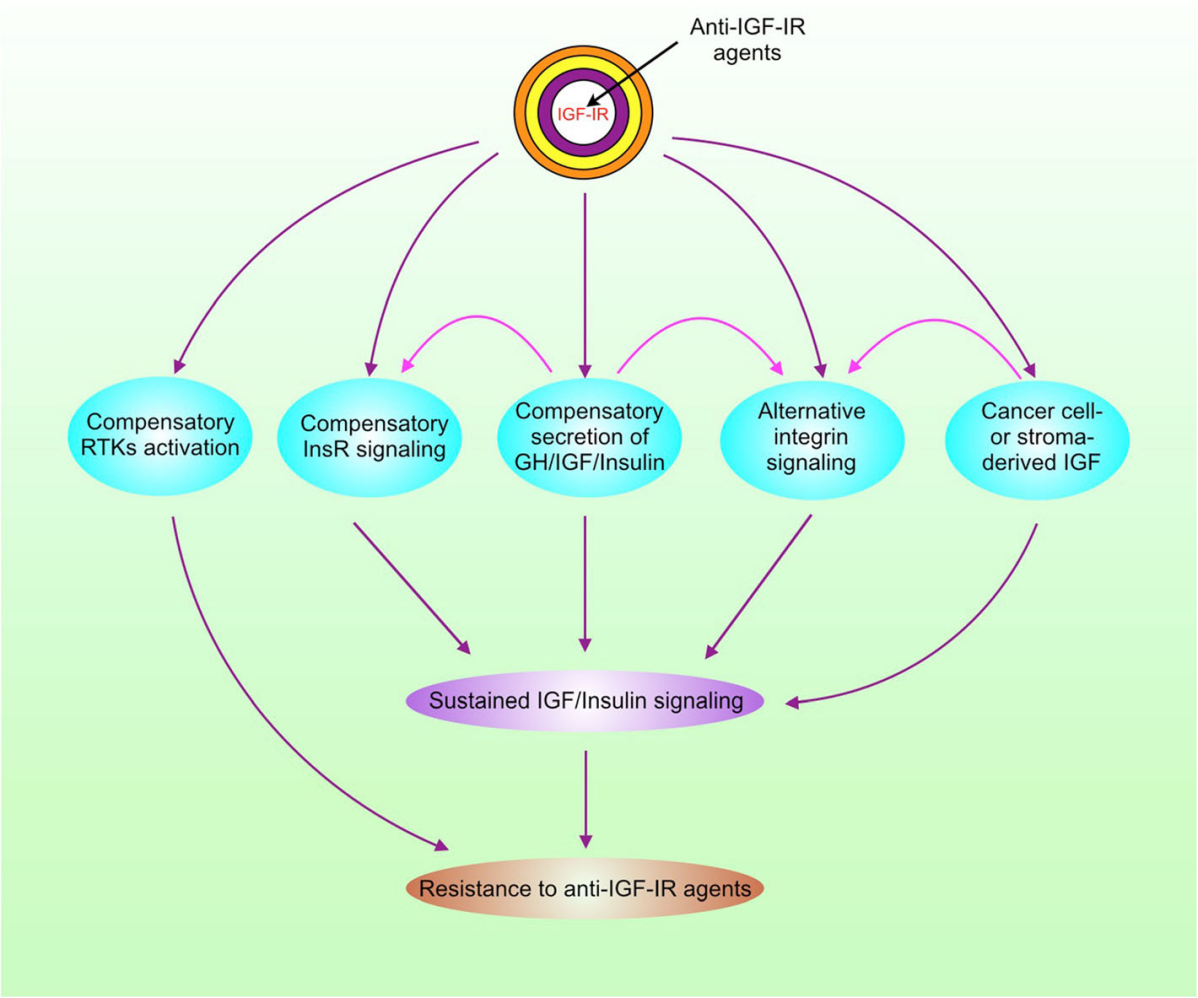
The mechanisms for resistance to anti-IGF-IR agents. RTKs, receptor tyrosine kinases

The high failure rate of anti-IGF-IR mAb/inhibitor monotherapy or combinational therapy in clinical trials promotes the investigators to inspect the mechanisms underlying resistance to IGF-IR inhibition in cancer and revisit the strategies to target IGF-IR pathway. While mutations in the drug target are involved in the resistance to many molecularly targeted agents [164], mutations in IGF-IR are barely detected in cancer cells resistant to anti-IGF-IR agents. There are multiple alternative signaling pathways to relay IGF/insulin signaling after IGF-IR is inhibited (Fig. 3).

### Compensatory secretion of growth hormone/insulin and activation of InsR

While growth hormone stimulates IGF1 synthesis, activation of IGF-IR/InsR by IGF feeds back to inhibit growth hormone synthesis [27]. Therefore, IGF-IR inhibition may paradoxically lead to increased production of IGF1/2 and insulin. Both IGF-IR and InsR-A are tumorigenic and responsive to IGF/insulin [165, 166]. Upon the prevention of IGF-IR ligand-binding and activation by anti-IGF-IR mAb, InsR may replace IGF-IR to bind IGF2 and insulin, leading to a compensatory increase in InsR phosphorylation/activation and sustained IGF/insulin signaling. Preclinical study demonstrates that overexpression of InsR-A in tumor cells confers complete resistance to cixutumumab, whereas InsR-B overexpression induces a partial resistance [167]. High InsR/IGF-IR ratios are associated with resistance to IGF-IR inhibition in human breast cancer cells [168]. In a pancreatic neuroendocrine cancer model, InsR knockout tumors are suppressed by anti-IGF-IR therapy, whereas InsR-expressed tumors are resistant to this therapy [168]. Among various types of human cancer, Ewing sarcoma is a promising indication for IGF-IR-targeted therapy, since some Ewing sarcomas are highly sensitive to IGF-IR inhibition. InsR-A confers both intrinsic and acquired resistance to anti-IGF-IR therapies as well [169]. Simultaneous inhibition of IGF-IR and InsR may provide superior antitumor efficacy compared with targeting IGF-IR alone. These studies lay a foundation for developing dual IGF-IR/InsR inhibitors such as BMS-754807 and linsitinib (OSI-906).

### Alternative integrin signaling

IGF not only binds to IGF-IR/InsR, but also directly binds to other receptors such as integrin, a family of adhesive receptors that promotes cancer stemness and cell survival to adapt to environmental and therapeutic stresses [170, 171]. When IGF1 binding to IGF-IR/InsR is blocked by the anti-IGF-IR mAb cixutumumab, IGF1 binding to integrin αvβ3 increases, resulting in activation of αvβ3 and its downstream effectors Src, FAK, and PI3K, which confers cixutumumab resistance [172]. Inhibition of integrin β3 or Src can improve the anti-cancer activity of cixutumumab in cixutumumab-resistant cell lines and patient-derived tumors [172]. Actually, a phase 1/2 clinical trial of combining ganitumab with Src inhibitor dasatinib in patients with embryonal and alveolar rhabdomyosarcoma is ongoing (Table 1). In addition, treatment of rhabdomyosarcoma cells with anti-IGF-IR mAb or IGF-IR/IR kinase inhibitor leads to increased activation of YES/Src family tyrosine kinase (SFK) [173]. Combined treatment with anti-IGF-IR agents and SFK inhibitors enhances antitumor activity [173].

### Alternative receptor tyrosine kinases activation

The receptor tyrosine kinase family composes of many collaborative members such as EGFR, ErbB2/3/4, MET, Axl, PDGF receptor (PDGFR), and IGF-IR, which share some effectors including PI3K/Akt/mTOR, Ras/Raf/Mek/Erk, Src, and JAK/STAT. Inhibition of one member of RTKs usually triggers the compensation by other members. While active IGF-IR can compensate EGFR inhibition, activation of the EGFR pathway also contributes to anti-IGF-IR drugs resistance in cancer [174, 175]. Of note, the adaptor protein IRS1 not only binds to IGF-IR, but also interacts with EGFR/ErbB3. Inhibition of IGF-IR may enhance ErbB3-IRS1 interaction [176]. Moreover, ErbB2 can form heterodimers with IGF-IR in IGF-IR inhibitor-resistant cancer cells, leading to the induction of ErbB2 phosphorylation by IGF2 [177]. Similar to ErbB2, the RON receptor tyrosine kinase interacts with IGF-IR and confers resistance to IGF-IR inhibitor in childhood sarcoma [178]. In addition, PDGFRα/β amplification, overexpression, and constitutive activation contribute to anti-IGF-IR mAb resistance [179, 180]. Therefore, the activation of PI3K/Akt, Ras, and STAT3 by other RTKs may confer resistance to IGF-IR inhibitors. In fact, upregulation of IRS1, PI3K, STAT3, and p38 MAPK is involved in resistance to dalotuzumab and linsitinib [128]. The bispecific antibody targeting both IGF-IR and EGFR holds promise in treating cancer, since it inhibits tumor growth and metastasis in preclinical studies [181]. As aforementioned, EMT is tightly linked to cancer stemness, metastasis, and drug resistance. Tumor cells with mesenchymal phenotype are resistant to the dual IGF-IR/InsR inhibitor linsitinib, due to the decreased expression of IGF2 and InsR, and phosphorylation of IGF-IR/InsR in these cells [182]. Although IGF-IR promotes EMT, it does not necessarily mean that inhibition of IGF-IR is able to reverse this phenotype, due to the compensation by other RTKs.

### Tumor microenvironment remodeling

Tumor cells reside in a highly heterogeneous niche composing of extracellular matrix and stromal cells such as fibroblasts, macrophages, and endothelial cells. Both fibroblasts and macrophages can be friends or foes of tumor cells, depending on their subtypes that usually co-exist in tumors to different extent [183]. Compared to the M1 macrophages that are pro-inflammatory and tumor suppressive, the anti-inflammatory and pro-tumor M2 macrophages are more abundant in tumors [184]. A rich tumor microenvironment supports tumor cells proliferation, survival, migration and invasion. On the other hand, tumor cells also secret cytokines to recruit and educate macrophages into M2 type, and stimulate stromal cells proliferation [185]. The tumor microenvironment is involved in acquired drug resistance that eventually impedes cancer therapy [186]. Tumor-associated macrophages and fibroblasts may secrete IGF to activate IGF-IR in cancer cells thereby promoting chemoresistance and radioresistance [187, 188]. In addition, dendritic cells in the tumor microenvironment can secret IGF and support T-ALL growth by activating IGF-IR [189]. Given that IGF can bypass IGF-IR to activate alternative signaling pathways, the stroma-derived IGF may contribute to resistance to anti-IGF-IR agents. On the other hand, treatment of cancer cells with the anti-IGF-IR mAb cixutumumab paradoxically triggers cancer cells to produce IGF2, which in turn recruits macrophages and fibroblasts to promote angiogenesis and metastasis thereby hampering the efficacy of cixutumumab [190]. Together, it is clear that anti-IGF-IR agents may paradoxically stimulate tumor microenvironment remodeling to boost IGF signaling through alternative pathways, leading to cancer resistance to anti-IGF-IR agents.

## Conclusion and perspectives

Mounting evidence demonstrates that receptor tyrosine kinases play important roles in carcinogenesis and tumor progression. Even with the remarkably good response to EGFR/ErbB2 inhibitors in some types of human cancer, the majority of patients eventually develop drug resistance, in which IGF-IR is also involved. Although it is clear that IGF-IR confers resistance to molecularly targeted therapy, chemotherapy and radiotherapy, the complex IGF biology is a great challenge for targeting this pathway in cancer. Either intrinsic or adaptive resistance to anti-IGF-IR agents is a troublesome roadblock. The high degree of tumor heterogeneity and adaptive cellular signaling plasticity, selection pressure, and clonal evolution substantially contribute to the resistance to molecularly targeted therapy including anti-IGF/IGF-IR agents [191].

The efficacy of molecularly targeted therapeutics may be highly selective in cancer patients. However, most of the clinical trials of anti-IGF-IR agents are conducted in unselected cancer patients. There are responders and non-responders in multiple clinical trials of IGF-IR inhibitors. While identification of the different molecular signatures and genotypes among responders and non-responders may help develop predictive biomarkers to enable patient stratification in clinical setting, the molecular and genetic heterogeneity in many tumors is still a big challenge for successful cancer therapy [182]. Nevertheless, identification of potential predictive biomarkers is an urgent task to advance the anti-IGF-IR therapeutics. The use of integrative proteomic-genomic techniques to analyze the samples of patients may be a reliable strategy. Preclinical studies have suggested potential biomarkers such as circulating free IGF levels, mutations of p53, BRCA1, KRAS and BRAF, IRS2 copy number gain, InsR/IGF-IR overexpression, InsR/IGF-IR pathway activation, PTEN loss, CD24, mesenchymal markers, β-catenin/TCF activity, FUS-DDIT3 fusion, and ERG [192–201]. Another study indicates that exclusive nuclear localization of IGF-IR is associated with better response to anti-IGF-IR antibody in patients with sarcomas [79]. These biomarkers should be evaluated in the clinic. Stratification of anti-IGF-IR agent responders according to molecular subtype remains to be a future direction.

Learning from the lessons of clinical trials, we need to adjust the strategy to target the complex IGF/IGF-IR signaling. Given the collaboration and compensation among different signaling pathways, IGF-IR and InsR should not be the sole targets to be blocked. IGF-IR-cotargeted multikinase inhibitors are preferable to treat cancer. The multikinase (FAK/IGF-IR) inhibitor CT-707 has anti-cancer effect in preclinical models and is subject to phase I clinical trial (clinicaltrials.gov, NCT02695550) [49]. In addition, the 4-aminopyrazolo[3,4-d]pyrimidine-based dual IGF-IR/Src inhibitor LL28 inhibits tumor growth in preclinical models [202]. We look forward to evaluating the efficacy of these multikinase inhibitors in clinical trials. Recently, the FDA-approved ALK inhibitor ceritinib has been identified as a multikinase inhibitor targeting IGF-IR/InsR, FAK, and RSK1/2 as well [203]. A recent study also demonstrates that ceritinib inhibits InsR phosphorylation and induces tumor regression in a pediatric patient with an unclassified brain tumor [204]. Another study suggests that IGF-IR is as an attractive target for treating patients with BCOR-alternated high-grade neuroepithelial tumor of the central nervous system [205]. It is worthwhile to ensure whether ceritinib can be repurposed to treat IGF-IR-activated cancer. In addition, the combined treatment with IGF-IR/InsR and CDK inhibitors warrants clinical evaluation.

Rather than targeting IGF-IR/InsR themselves, an alternative strategy would be inhibition of the master regulators of RTKs or downstream signaling hubs such as IRS1/2 [205–208]. Moreover, IGF-IR has kinase-independent functions, such as nuclear translocation and regulation of gene expression, and interaction with SGLT1 to maintain intracellular glucose levels [209]. These kinase-independent functions of IGF-IR are another challenge to treat cancer with anti-IGF-IR agents. Inhibition of the kinase activity of IGF-IR may be not enough to eliminate its pro-tumor effects. Targeting InsR/IGF-IR expression or degradation should be considered as another strategy awaiting further exploration [210, 211]. Given that inhibition of IGF-IR may paradoxically lead to upregulation of IGF1/2 expression, and IGF1/2 have IGF-IR/InsR-independent functions, it warrants to investigate whether co-targeting IGF and IGF-IR/InsR-A may achieve objective responses.

## Data Availability

Not applicable.

## Abbreviations

ALK: Anaplastic lymphoma kinase
ALL: Acute lymphoblastic leukemia
EGF: Epidermal growth factor
ER: Estrogen receptor
FAK: Focal adhesion kinase
FGF: Fibroblast growth factor
HGF: Hepatocyte growth factor
HNSCC: Head and neck squamous carcinoma
IGF: Insulin-like growth factor
IGFBP: IGF-binding protein
IGF-IR: Type I insulin-like growth factor receptor
IGF-IIR: Type II IGF receptor
InsR: Insulin receptor
mAbs: Monoclonal antibodies
MAPK: Mitogen-activated protein kinase
PDGF: Platelet-derived growth factor
PI3K: Phosphatidylinositol 3-kinase
RTK: Receptor tyrosine kinase
TGF: Transforming growth factor
VEGF: Vascular endothelial growth factor

## References

[1] Bach LA (2018). IGF-binding proteins. J Mol Endocrinol.

[2] Roddam AW, Allen NE, Appleby P, Key TJ, Ferrucci L, Carter HB (2008). Insulin-like growth factors, their binding proteins, and prostate cancer risk: analysis of individual patient data from 12 prospective studies. Ann Intern Med.

[3] Price AJ, Allen NE, Appleby PN, Crowe FL, Travis RC, Tipper SJ (2012). Insulin-like growth factor-I concentration and risk of prostate cancer: results from the European Prospective Investigation into Cancer and Nutrition. Cancer Epidemiol Biomark Prev.

[4] Ho GYF, Zheng SL, Cushman M, Perez-Soler R, Kim M, Xue X (2016). Associations of insulin and IGFBP-3 with lung cancer susceptibility in current smokers. J Natl Cancer Inst.

[5] Yoon YS, Keum N, Zhang X, Cho E, Giovannucci EL (2015). Circulating levels of IGF-1, IGFBP-3, and IGF-1/IGFBP-3 molar ratio and colorectal adenomas: a meta-analysis. Cancer Epidemiol.

[6] Adachi Y, Nojima M, Mori M, Yamashita K, Yamano HO, Nakase H (2017). Insulin-like growth factor-1, IGF binding protein-3, and the risk of esophageal cancer in a nested case-control study. World J Gastroenterol.

[7] Adachi Y, Nojima M, Mori M, Kubo T, Yamano HO, Lin Y (2019). Circulating insulin-like growth factor binding protein-3 and risk of gastrointestinal malignant tumors. J Gastroenterol Hepatol.

[8] Lin C, Travis RC, Appleby PN, Tipper S, Weiderpass E, Chang-Claude J (2018). Pre-diagnostic circulating insulin-like growth factor-I and bladder cancer risk in the European Prospective Investigation into Cancer and Nutrition. Int J Cancer.

[9] Perez-Cornago A, Appleby PN, Tipper S (2017). Prediagnostic circulating concentrations of plasma insulin-like growth factor-I and risk of lymphoma in the European Prospective Investigation into Cancer and Nutrition. Int J Cancer.

[10] Bradbury KE, Appleby PN, Tipper SJ, Key TJ, Allen NE, Nieters A (2019). Circulating insulin-like growth factor I in relation to melanoma risk in the European Prospective Investigation into Cancer and Nutrition. Int J Cancer.

[11] Chng WJ, Gualberto A, Fonseca R (2006). IGF-1R is overexpressed in poor-prognostic subtypes of multiple myeloma. Leuk Res.

[12] Sprynski AC, Hose D, Kassambara A, Vincent L, Jourdan M, Rossi JF (2010). Insulin is a potent myeloma cell growth factor through insulin/IGF-1 hybrid receptor activation. Leuk Res.

[13] Vishwamitra D, George SK, Shi P, Kaseb AO, Amin HM (2017). Type I insulin-like growth factor receptor signaling in hematological malignancies. Oncotarget..

[14] Xie J, Chen X, Zheng J, Li C, Stacy S, Holzenberger M (2015). IGF-IR determines the fates of BCR/ABL leukemia. J Hematol Oncol.

[15] Medyouf H, Gusscott S, Wang H, Tseng JC, Wai C, Nemirovsky O (2011). High-level IGF1R expression is required for leukemia-initiating cell activity in T-ALL and is supported by notch signaling. J Exp Med.

[16] Lakshmikuttyamma A, Pastural E, Takahashi N, Sawada K, Sheridan DP, DeCoteau JF (2008). Bcr-Abl induces autocrine IGF-1 signaling. Oncogene..

[17] Doepfner KT, Spertini O, Arcaro A (2007). Autocrine insulin-like growth factor-I signaling promotes growth and survival of human acute myeloid leukemia cells via the phosphoinositide 3-kinase/Akt pathway. Leukemia..

[18] Yaktapour N, Übelhart R, Schüler J, Aumann K, Dierks C, Burger M (2013). Insulin-like growth factor-1 receptor (IGF1R) as a novel target in chronic lymphocytic leukemia. Blood..

[19] Shi P, Lai R, Lin Q, Iqbal AS, Young LC, Kwak LW (2009). IGF-IR tyrosine kinase interacts with NPM-ALK oncogene to induce survival of T-cell ALK+ anaplastic large-cell lymphoma cells. Blood..

[20] Mitsiades CS, Mitsiades NS, McMullan CJ, Poulaki V, Shringarpure R, Akiyama M (2004). Inhibition of the insulin-like growth factor receptor-1 tyrosine kinase activity as a therapeutic strategy for multiple myeloma, other hematologic malignancies, and solid tumors. Cancer Cell.

[21] Maris C, D’Haene N, Trépant AL, Le Mercier M, Sauvage S, Allard J (2015). IGF-IR: a new prognostic biomarker for human glioblastoma. Br J Cancer.

[22] Khatib AM, Siegfried G, Prat A, Luis J, Chrétien M, Metrakos P (2001). Inhibition of proprotein convertases is associated with loss of growth and tumorigenicity of HT-29 human colon carcinoma cells: importance of insulin-like growth factor-1 (IGF-1) receptor processing in IGF-1-mediated functions. J Biol Chem.

[23] LeRoith D, Werner H, Beitner-Johnson D, Roberts CT (1995). Molecular and cellular aspects of the insulin-like growth factor I receptor. Endocr Rev.

[24] Ullrich A, Gray A, Tam AW, Yang-Feng T, Tsubokawa M, Collins C (1986). Insulin-like growth factor I receptor primary structure: comparison with insulin receptor suggests structural determinants that define functional specificity. EMBO J.

[25] Seino S, Bell GI (1989). Alternative splicing of human insulin receptor messenger RNA. Biochem Biophys Res Commun.

[26] Evdokimova V, Tognon CE, Benatar T, Yang W, Krutikov K, Pollak M (2012). IGFBP7 binds to the IGF-1 receptor and blocks its activation by insulin-like growth factors. Sci Signal.

[27] Yee D (2018). Anti-insulin-like growth factor therapy in breast cancer. J Mol Endocrinol.

[28] Taniguchi CM, Emanuelli B, Kahn CR (2006). Critical nodes in signalling pathways: insights into insulin action. Nat Rev Mol Cell Biol.

[29] Liu D, Rutter WJ, Wang LH (1993). Modulating effects of the extracellular sequence of the human insulin-like growth factor I receptor on its transforming and tumorigenic potential. J Virol.

[30] Lee JH, Choi SI, Kim RK, Cho EW, Kim IG (2018). Tescalcin/c-Src/IGF1Rβ-mediated STAT3 activation enhances cancer stemness and radioresistant properties through ALDH1. Sci Rep.

[31] Tahimic CG, Long RK, Kubota T, Sun MY, Elalieh H, Fong C (2016). Regulation of ligand and shear stress-induced insulin-like growth factor 1 (IGF1) signaling by the integrin pathway. J Biol Chem.

[32] Yin Y, Hua H, Li M, Liu S, Kong Q, Shao T (2016). mTORC2 promotes type I insulin-like growth factor receptor and insulin receptor activation through the tyrosine kinase activity of mTOR. Cell Res.

[33] De Meyts P, Shymko RM (2000). Timing-dependent modulation of insulin mitogenic versus metabolic signalling. Novartis Found Symp.

[34] Accili D, Drago J, Lee EJ, Johnson MD, Cool MH, Salvatore P (1996). Early neonatal death in mice homozygous for a null allele of the insulin receptor gene. Nat Genet.

[35] Farabaugh SM, Boone DN, Lee AV (2015). Role of IGF1R in breast cancer subtypes, stemness, and lineage differentiation. Front Endocrinol (Lausanne).

[36] Peiró G, Adrover E, Sánchez-Tejada L, Lerma E, Planelles M, Sánchez-Payá J (2011). Increased insulin-like growth factor-1 receptor mRNA expression predicts poor survival in immunophenotypes of early breast carcinoma. Mod Pathol.

[37] Law JH, Habibi G, Hu K, Masoudi H, Wang MY, Stratford AL (2008). Phosphorylated insulin-like growth factor-i/insulin receptor is present in all breastcancer subtypes and is related to poor survival. Cancer Res.

[38] Creighton CJ, Casa A, Lazard Z, Huang S, Tsimelzon A, Hilsenbeck SG (2008). Insulin-like growth factor-I activates gene transcription programs strongly associated with poor breast cancer prognosis. J Clin Oncol.

[39] Yerushalmi R, Gelmon KA, Leung S, Gao D, Cheang M, Pollak M (2012). Insulin-like growth factor receptor (IGF-1R) in breast cancer subtypes. Breast Cancer Res Treat.

[40] Mountzios G, Aivazi D, Kostopoulos I, Goldbogen J, Southall B (2014). Differential expression of the insulin-like growth factor receptor among early breast cancer subtypes. PLoS One.

[41] Aleksic T, Verrill C, Bryant RJ, Han C, Worrall AR, Brureau L (2017). IGF-1R associates with adverse outcomes after radical radiotherapy for prostate cancer. Br J Cancer.

[42] Dale OT, Aleksic T, Shah KA, Han C, Mehanna H, Rapozo DC (2015). IGF-1R expression is associated with HPV-negative status and adverse survival in head and neck squamous cell cancer. Carcinogenesis..

[43] Moreno-Acosta P, Vallard A, Carrillo S, Gamboa O, Romero-Rojas A, Molano M (2017). Biomarkers of resistance to radiation therapy: a prospective study in cervical carcinoma. Radiat Oncol.

[44] Fruman DA, Chiu H, Hopkins BD, Bagrodia S, Cantley LC, Abraham RT (2017). The PI3K pathway in human disease. Cell..

[45] Giorgetti S, Ballotti R, Kowalski-Chauvel A, Tartare S, Van Obberghen E (1993). The insulin and insulin-like growth factor-I receptor substrate IRS-1 associates with and activates phosphatidylinositol 3-kinase in vitro. J Biol Chem.

[46] Hua H, Kong Q, Zhang H, Wang J, Luo T, Jiang Y (2019). Targeting mTOR for cancer therapy. J Hematol Oncol.

[47] Nitulescu GM, Van De Venter M, Nitulescu G, Nitulescu G, Ungurianu A, Juzenas P (2018). The Akt pathway in oncology therapy and beyond. Int J Oncol.

[48] Qin X, Li J, Sun J, Liu L, Chen D, Liu Y (2019). Low shear stress induces ERK nuclear localization and YAP activation to control the proliferation of breast cancer cells. Biochem Biophys Res Commun.

[49] Zhu H, Wang DD, Yuan T, Yan FJ, Zeng CM, Dai XY (2018). Multikinase inhibitor CT-707 targets liver cancer by interrupting the hypoxia-activated IGF-1R-YAP axis. Cancer Res.

[50] Straßburger K, Tiebe M, Pinna F, Breuhahn K, Teleman AA (2012). Insulin/IGF signaling drives cell proliferation in part via Yorkie/YAP. Dev Biol.

[51] Ravid D, Maor S, Werner H, Liscovitch M (2005). Caveolin-1 inhibits cell detachment-induced p53 activation and anoikis by upregulation of insulin-like growth factor-I receptors and signaling. Oncogene..

[52] Zhang W, Zong CS, Hermanto U, Lopez-Bergami P, Ronai Z, Wang LH (2006). RACK1 recruits STAT3 specifically to insulin and insulin-like growth factor 1 receptors for activation, which is important for regulating anchorage-independent growth. Mol Cell Biol.

[53] Kiely PA, Sant A, O’Connor R (2002). RACK1 is an insulin-like growth factor 1 (IGF-1) receptor-interacting protein that can regulate IGF-1-mediated Akt activation and protection from cell death. J Biol Chem.

[54] Li H, Baldwin BR, Zahnow CA (2011). LIP expression is regulated by IGF-1R signaling and participates in suppression of anoikis. Mol Cancer.

[55] Sachdev D, Zhang X, Matise I, Gaillard-Kelly M, Yee D (2010). The type I insulin-like growth factor receptor regulates cancer metastasis independently of primary tumor growth by promoting invasion and survival. Oncogene..

[56] Varkaris A, Gaur S, Parikh NU, Song JH, Dayyani F, Jin JK (2013). Ligand-independent activation of MET through IGF-1/IGF-1R signaling. Int J Cancer.

[57] Jaquish DV, Yu PT, Shields DJ, French RP, Maruyama KP, Niessen S (2011). IGF1-R signals through the RON receptor to mediate pancreatic cancer cell migration. Carcinogenesis..

[58] Svalina MN, Kikuchi K, Abraham J, Lal S, Davare MA, Settelmeyer TP (2016). IGF1R as a key target in high risk, metastatic medulloblastoma. Sci Rep.

[59] Vella V, Malaguarnera R, Nicolosi ML, Morrione A, Belfiore A (2019). Insulin/IGF signaling and discoidin domain receptors: an emerging functional connection. Biochim Biophys Acta, Mol Cell Res.

[60] Azizi R, Salemi Z, Fallahian F, Aghaei M (2019). Inhibition of didscoidin domain receptor 1 reduces epithelial-mesenchymal transition and induce cell-cycle arrest and apoptosis in prostate cancer cell lines. J Cell Physiol.

[61] Cheng J, He S, Wang M, Zhou L, Zhang Z, Feng X (2019). The caspase-3/PKCδ/Akt/VEGF-A signaling pathway mediates tumor repopulation during radiotherapy. Clin Cancer Res.

[62] Datta K, Nambudripad R, Pal S, Zhou M, Cohen HT, Mukhopadhyay D (2000). Inhibition of insulin-like growth factor-I-mediated cell signaling by the von Hippel-Lindau gene product in renal cancer. J Biol Chem.

[63] Lyons A, Coleman M, Riis S, Favre C, O’Flanagan CH, Zhdanov AV (2017). Insulin-like growth factor 1 signaling is essential for mitochondrial biogenesis and mitophagy in cancer cells. J Biol Chem.

[64] Yang Y, Yee D. IGF-I regulates redox status in breast cancer cells by activating the amino acid transport molecule xC-. Cancer Res 2014;74:2295-305. 10.1158/0008-5472.CAN-13-1803 PMC4006361 24686172

[65] Logan S, Pharaoh GA, Marlin MC, Masser DR, Matsuzaki S, Wronowski B (2018). Insulin-like growth factor receptor signaling regulates working memory, mitochondrial metabolism, and amyloid-β uptake in astrocytes. Mol Metab.

[66] Li H, Batth IS, Qu X, Xu L, Song N, Wang R (2017). IGF-IR signaling in epithelial to mesenchymal transition and targeting IGF-IR therapy: overview and new insights. Mol Cancer.

[67] Taliaferro-Smith L, Oberlick E, Liu T, McGlothen T, Alcaide T, Tobin R (2015). FAK activation is required for IGF1R-mediated regulation of EMT, migration, and invasion in mesenchymal triple negative breast cancer cells. Oncotarget..

[68] Kim HJ, Litzenburger BC, Cui X, Delgado DA, Grabiner BC, Lin X (2007). Constitutively active type I insulin-like growth factor receptor causes transformation and xenograft growth of immortalized mammary epithelial cells and is accompanied by an epithelial-to-mesenchymal transition mediated by NF-kappaB and snail. Mol Cell Biol.

[69] Ye X, Tam WL, Shibue T, Kaygusuz Y, Reinhardt F, Ng Eaton E (2015). Distinct EMT programs control normal mammary stem cells and tumour-initiating cells. Nature..

[70] Yao C, Su L, Shan J, Zhu C, Liu L, Liu C (2016). IGF/STAT3/NANOG/Slug signaling axis simultaneously controls epithelial-mesenchymal transition and stemness maintenance in colorectal cancer. Stem Cells.

[71] Tominaga K, Shimamura T, Kimura N, Murayama T, Matsubara D, Kanauchi H (2017). Addiction to the IGF2-ID1-IGF2 circuit for maintenance of the breast cancer stem-like cells. Oncogene..

[72] Xu C, Xie D, Yu SC, Yang XJ, He LR, Yang J (2013). β-Catenin/POU5F1/SOX2 transcription factor complex mediates IGF-I receptor signaling and predicts poor prognosis in lung adenocarcinoma. Cancer Res.

[73] Chang WW, Lin RJ, Yu J, Chang WY, Fu CH, Lai A (2013). The expression and significance of insulin-like growth factor-1 receptor and its pathway on breast cancer stem/progenitors. Breast Cancer Res.

[74] Steder M, Alla V, Meier C, Spitschak A, Pahnke J, Fürst K (2013). DNp73 exerts function in metastasis initiation by disconnecting the inhibitory role of EPLIN on IGF1R-AKT/STAT3 signaling. Cancer Cell.

[75] Meier C, Hardtstock P, Joost S, Alla V, Pützer BM (2016). p73 and IGF1R regulate emergence of aggressive cancer stem-like features via miR-885-5p control. Cancer Res.

[76] Chen WJ, Ho CC, Chang YL, Chen HY, Lin CA, Ling TY (2014). Cancer-associated fibroblasts regulate the plasticity of lung cancer stemness via paracrine signalling. Nat Commun.

[77] Kim IG, Kim SY, Choi SI, Lee JH, Kim KC, Cho EW (2014). Fibulin-3-mediated inhibition of epithelial-to-mesenchymal transition and self-renewal of ALDH+ lung cancer stem cells through IGF1R signaling. Oncogene..

[78] Aleksic T, Chitnis MM, Perestenko OV (2010). Type 1 insulin-like growth factor receptor translocates to the nucleus of human tumor cells. Cancer Res.

[79] Asmane I, Watkin E, Alberti L, Gao S, Thomas PH, Turner GD (2012). Insulin-like growth factor type 1 receptor (IGF-1R) exclusive nuclear staining: a predictive biomarker for IGF-1R monoclonal antibody (Ab) therapy in sarcomas. Eur J Cancer.

[80] Aslam MI, Hettmer S, Abraham J, Latocha D, Soundararajan A, Huang ET (2013). Dynamic and nuclear expression of PDGFRα and IGF-1R in alveolar rhabdomyosarcoma. Mol Cancer Res.

[81] Sehat B, Tofigh A, Lin Y, Trocmé E, Liljedahl U, Lagergren J (2010). SUMOylation mediates the nuclear translocation and signaling of the IGF-1 receptor. Sci Signal.

[82] Deng H, Lin Y, Badin M (2011). Over-accumulation of nuclear IGF-1 receptor in tumor cells requires elevated expression of the receptor and the SUMO-conjugating enzyme Ubc9. Biochem Biophys Res Commun.

[83] Packham S, Warsito D, Lin Y, Vasilcanu D, Strömberg T, Jernberg-Wiklund H (2015). Nuclear translocation of IGF-1R via p150(Glued) and an importin-β/RanBP2-dependent pathway in cancer cells. Oncogene..

[84] Guerard M, Robin T, Perron P, Hatat AS, David-Boudet L, Vanwonterghem L (2018). Nuclear translocation of IGF1R by intracellular amphiregulin contributes to the resistance of lung tumour cells to EGFR-TKI. Cancer Lett.

[85] Sarfstein R, Pasmanik-Chor M, Yeheskel A, Edry L, Shomron N, Warman N (2012). Insulin-like growth factor-I receptor (IGF-IR) translocates to nucleus and autoregulates IGF-IR gene expression in breast cancer cells. J Biol Chem.

[86] Aleksic T, Gray N, Wu X, Rieunier G, Osher E, Mills J (2018). Nuclear IGF1R interacts with regulatory regions of chromatin to promote RNA polymerase II recruitment and gene expression associated with advanced tumor stage. Cancer Res.

[87] Warsito D, Sjöström S, Andersson S, Larsson O, Sehat B (2012). Nuclear IGF1R is a transcriptional co-activator of LEF1/TCF. EMBO Rep.

[88] Jamwal G, Singh G, Dar MS, Singh P, Bano N, Syed SH (1865). Identification of a unique loss-of-function mutation in IGF1R and a crosstalk between IGF1R and Wnt/β-catenin signaling pathways. Biochim Biophys Acta, Mol Cell Res.

[89] Waraky A, Lin Y, Warsito D, Haglund F, Aleem E, Larsson O (2017). Nuclear insulin-like growth factor 1 receptor phosphorylates proliferating cell nuclear antigen and rescues stalled replication forks after DNA damage. J Biol Chem.

[90] Warsito D, Lin Y, Gnirck AC, Sehat B, Larsson O (2016). Nuclearly translocated insulin-like growth factor 1 receptor phosphorylates histone H3 at tyrosine 41 and induces SNAI2 expression via Brg1 chromatin remodeling protein. Oncotarget..

[91] Heskamp S, Boerman OC, Molkenboer-Kuenen JD, Wauters CA, Strobbe LJ, Mandigers CM (2015). Upregulation of IGF-1R expression during neoadjuvant therapy predicts poor outcome in breast cancer patients. PLoS One.

[92] Chitnis MM, Lodhia KA, Aleksic T, Gao S, Protheroe AS, Macaulay VM (2014). IGF-1R inhibition enhances radiosensitivity and delays double-strand break repair by both non-homologous end-joining and homologous recombination. Oncogene..

[93] Szymonowicz K, Oeck S, Krysztofiak A, van der Linden J, Iliakis G, Jendrossek V (2018). Restraining Akt1 phosphorylation attenuates the repair of radiation-induced DNA double-strand breaks and reduces the survival of irradiated cancer cells. Int J Mol Sci.

[94] Chen YH, Wang CW, Wei MF, Tzeng YS, Lan KH, Cheng AL (2019). Maintenance BEZ235 treatment prolongs the therapeutic effect of the combination of BEZ235 and radiotherapy for colorectal cancer. Cancers (Basel).

[95] Lodhia KA, Gao S, Aleksic T, Esashi F, Macaulay VM (2015). Suppression of homologous recombination sensitizes human tumor cells to IGF-1R inhibition. Int J Cancer.

[96] O’Flanagan CH, O’Shea S, Lyons A, Fogarty FM, McCabe N, Kennedy RD (2016). IGF-1R inhibition sensitizes breast cancer cells to ATM-related kinase (ATR) inhibitor and cisplatin. Oncotarget..

[97] Ferté C, Loriot Y, Clémenson C, Commo F, Gombos A, Bibault JE (2013). IGF-1R targeting increases the antitumor effects of DNA-damaging agents in SCLC model: an opportunity to increase the efficacy of standard therapy. Mol Cancer Ther.

[98] Flanigan SA, Pitts TM, Eckhardt SG, Tentler JJ, Tan AC, Thorburn A (2010). The insulin-like growth factor I receptor/insulin receptor tyrosine kinase inhibitor PQIP exhibits enhanced antitumor effects in combination with chemotherapy against colorectal cancer models. Clin Cancer Res.

[99] Zeng X, Zhang H, Oh A, Zhang Y, Yee D (2012). Enhancement of doxorubicin cytotoxicity of human cancer cells by tyrosine kinase inhibition of insulin receptor and type I IGF receptor. Breast Cancer Res Treat.

[100] Beauchamp MC, Knafo A, Yasmeen A, Carboni JM, Gottardis MM, Pollak MN (2009). BMS-536924 sensitizes human epithelial ovarian cancer cells to the PARP inhibitor, 3-aminobenzamide. Gynecol Oncol.

[101] Singh RK, Gaikwad SM, Jinager A, Chaudhury S, Maheshwari A, Ray P (2014). IGF-1R inhibition potentiates cytotoxic effects of chemotherapeutic agents in early stages of chemoresistant ovarian cancer cells. Cancer Lett.

[102] Ramcharan R, Aleksic T, Kamdoum WP, Gao S, Pfister SX, Tanner J (2015). IGF-1R inhibition induces schedule-dependent sensitization of human melanoma to temozolomide. Oncotarget..

[103] Dallas NA, Xia L, Fan F, Gray MJ, Gaur P, van Buren G (2009). Chemoresistant colorectal cancer cells, the cancer stem cell phenotype, and increased sensitivity to insulin-like growth factor-I receptor inhibition. Cancer Res.

[104] Camblin AJ, Pace EA, Adams S, Curley MD, Rimkunas V, Nie L (2018). Dual Inhibition of IGF-1R and ErbB3 enhances the activity of gemcitabine and Nab-paclitaxel in preclinical models of pancreatic cancer. Clin Cancer Res.

[105] Kuhn DJ, Berkova Z, Jones RJ, Woessner R, Bjorklund CC, Ma W (2012). Targeting the insulin-like growth factor-1 receptor to overcome bortezomib resistance in preclinical models of multiple myeloma. Blood..

[106] Vaira V, Lee CW, Goel HL, Bosari S, Languino LR, Altieri DC (2007). Regulation of survivin expression by IGF-1/mTOR signaling. Oncogene..

[107] Fox EM, Miller TW, Balko JM, Kuba MG, Sánchez V, Smith RA (2011). A kinome-wide screen identifies the insulin/IGF-I receptor pathway as a mechanism of escape from hormone dependence in breast cancer. Cancer Res.

[108] Fox EM, Kuba MG, Miller TW, Davies BR, Arteaga CL (2013). Autocrine IGF-I/insulin receptor axis compensates for inhibition of AKT in ER-positive breast cancer cells with resistance to estrogen deprivation. Breast Cancer Res.

[109] Choi HJ, Joo HS, Won HY, Min KW, Kim HY, Son T, et al. Role of RBP2-induced ER and IGF1R-ErbB signaling in tamoxifen resistance in breast cancer. J Natl Cancer Inst. 2018;110. 10.1093/jnci/djx207. 10.1093/jnci/djx207 29028222

[110] Chakraborty A, Hatzis C, DiGiovanna MP (2017). Co-targeting the HER and IGF/insulin receptor axis in breast cancer, with triple targeting with endocrine therapy for hormone-sensitive disease. Breast Cancer Res Treat.

[111] Locke JA, Guns ES, Lehman ML, Ettinger S, Zoubeidi A, Lubik A (2010). Arachidonic acid activation of intratumoral steroid synthesis during prostate cancer progression to castration resistance. Prostate..

[112] Lubik AA, Gunter JH, Hollier BG, Ettinger S, Fazli L, Stylianou N (2013). IGF2 increases de novo steroidogenesis in prostate cancer cells. Endocr Relat Cancer.

[113] Fahrenholtz CD, Beltran PJ, Burnstein KL (2013). Targeting IGF-IR with ganitumab inhibits tumorigenesis and increases durability of response to androgen-deprivation therapy in VCaP prostate cancer xenografts. Mol Cancer Ther.

[114] Wang X, Huang Y, Christie A, Bowden M, Lee GS, Kantoff PW (2015). Cabozantinib inhibits abiraterone’s upregulation of IGFIR phosphorylation and enhances its anti-prostate cancer activity. Clin Cancer Res.

[115] Nordstrand A, Bergström SH, Thysell E, Bovinder-Ylitalo E, Lerner UH, Widmark A (2017). Inhibition of the insulin-like growth factor-1 receptor potentiates acute effects of castration in a rat model for prostate cancer growth in bone. Clin Exp Metastasis.

[116] Jia Y, Zhang Y, Qiao C, Liu G, Zhao Q, Zhou T (2013). IGF-1R and ErbB3/HER3 contribute to enhanced proliferation and carcinogenesis in trastuzumab-resistant ovarian cancer model. Biochem Biophys Res Commun.

[117] Codony-Servat J, Cuatrecasas M, Asensio E, Montironi C, Martínez-Cardús A, Marín-Aguilera M (2017). Nuclear IGF-1R predicts chemotherapy and targeted therapy resistance in metastatic colorectal cancer. Br J Cancer.

[118] Ma Y, Tang N, Thompson RC, Mobley BC, Clark SW, Sarkaria JN (2016). InsR/IGF1R pathway mediates resistance to EGFR inhibitors in glioblastoma. Clin Cancer Res.

[119] Vaquero J, Lobe C, Tahraoui S, Clapéron A, Mergey M, Merabtene F (2018). The IGF2/IR/IGF1R pathway in tumor cells and myofibroblasts mediates resistance to EGFR inhibition in cholangiocarcinoma. Clin Cancer Res.

[120] Vitiello PP, Cardone C, Martini G, Ciardiello D, Belli V, Matrone N (2019). Receptor tyrosine kinase-dependent PI3K activation is an escape mechanism to vertical suppression of the EGFR/RAS/MAPK pathway in KRAS-mutated human colorectal cancer cell lines. J Exp Clin Cancer Res.

[121] Sanderson MP, Hofmann MH, Garin-Chesa P, Schweifer N, Wernitznig A, Fischer S (2017). The IGF1R/INSR inhibitor BI 885578 selectively inhibits growth of IGF2-overexpressing colorectal cancer tumors and potentiates the efficacy of anti-VEGF therapy. Mol Cancer Ther.

[122] Tovar V, Cornella H, Moeini A, Vidal S, Hoshida Y, Sia D (2017). Tumour initiating cells and IGF/FGF signalling contribute to sorafenib resistance in hepatocellular carcinoma. Gut..

[123] Merino VF, Cho S, Liang X, Park S, Jin K, Chen Q (2017). Inhibitors of STAT3, β-catenin, and IGF-1R sensitize mouse PIK3CA-mutant breast cancer to PI3K inhibitors. Mol Oncol.

[124] Huo X, Liu S, Shao T, Hua H, Kong Q, Wang J (2014). GSK3 protein positively regulates type I insulin-like growth factor receptor through forkhead transcription factors FOXO1/3/4. J Biol Chem.

[125] Scheffold A, Jebaraj BMC, Tausch E, Bloehdorn J, Ghia P, Yahiaoui A (2019). IGF1R as druggable target mediating PI3K-δ inhibitor resistance in a murine model of chronic lymphocytic leukemia. Blood..

[126] Leroy C, Ramos P, Cornille K, Bonenfant D, Fritsch C, Voshol H (2016). Activation of IGF1R/p110β/AKT/mTOR confers resistance to α-specific PI3K inhibition. Breast Cancer Res.

[127] Zorea J, Prasad M, Cohen L, Elkabets M, Karabchevsky A (2018). IGF1R upregulation confers resistance to isoform-specific inhibitors of PI3K in PIK3CA-driven ovarian cancer. Cell Death Dis.

[128] Yoon SO, Shin S, Karreth FA, Buel GR, Jedrychowski MP, Plas DR (2017). Focal adhesion- and IGF1R-dependent survival and migratory pathways mediate tumor resistance to mTORC1/2 inhibition. Mol Cell.

[129] Lamhamedi-Cherradi SE, Menegaz BA, Ramamoorthy V, Vishwamitra D, Wang Y, Maywald RL (2016). IGF-1R and mTOR blockade: novel resistance mechanisms and synergistic drug combinations for Ewing sarcoma. J Natl Cancer Inst.

[130] Benito-Jardón L, Díaz-Martínez M, Arellano-Sánchez N, Vaquero-Morales P, Esparís-Ogando A, Teixidó J (2019). Resistance to MAPK inhibitors in melanoma involves activation of the IGF1R-MEK5-Erk5 pathway. Cancer Res.

[131] Strub T, Ghiraldini FG, Carcamo S, Li M, Wroblewska A, Singh R (2018). SIRT6 haploinsufficiency induces BRAF^V600E^ melanoma cell resistance to MAPK inhibitors via IGF signalling. Nat Commun.

[132] Guenther LM, Dharia NV, Ross L, Conway A, Robichaud AL, Catlett JL (2019). A combination CDK4/6 and IGF1R inhibitor strategy for Ewing sarcoma. Clin Cancer Res.

[133] Isozaki H, Ichihara E, Takigawa N, Ohashi K, Ochi N, Yasugi M (2016). Non-small cell lung cancer cells acquire resistance to the ALK Inhibitor alectinib by activating alternative receptor tyrosine kinases. Cancer Res.

[134] George B, George SK, Shi W, Haque A, Shi P, Eskandari G (2019). Dual inhibition of IGF-IR and ALK as an effective strategy to eradicate NPM-ALK^+^ T-cell lymphoma. J Hematol Oncol.

[135] Lovly CM, McDonald NT, Chen H, Ortiz-Cuaran S, Heukamp LC, Yan Y (2014). Rationale for co-targeting IGF-1R and ALK in ALK fusion-positive lung cancer. Nat Med.

[136] Xu Y, Vakoc CR (2017). Targeting cancer cells with BET bromodomain inhibitors. Cold Spring Harb Perspect Med.

[137] Li W, Gupta SK, Han W, Kundson RA, Nelson S, Knutson D (2019). Targeting MYC activity in double-hit lymphoma with MYC and BCL2 and/or BCL6 rearrangements with epigenetic bromodomain inhibitors. J Hematol Oncol.

[138] Stuhlmiller TJ, Miller SM, Zawistowski JS, Nakamura K, Beltran AS, Duncan JS (2015). Inhibition of lapatinib-induced kinome reprogramming in ERBB2-positive breast cancer by targeting BET family bromodomains. Cell Rep.

[139] Loganathan SN, Tang N, Holler AE, Wang N, Wang J (2019). Targeting the IGF1R/PI3K/AKT pathway sensitizes Ewing sarcoma to BET bromodomain inhibitors. Mol Cancer Ther.

[140] Kim KH, Roberts CW (2016). Targeting EZH2 in cancer. Nat Med.

[141] Kleer CG, Cao Q, Varambally S, Shen R, Ota I, Tomlins SA (2003). EZH2 is a marker of aggressive breast cancer and promotes neoplastic transformation of breast epithelial cells. Proc Natl Acad Sci U S A.

[142] Li B, Chng WJ (2019). EZH2 abnormalities in lymphoid malignancies: underlying mechanisms and therapeutic implications. J Hematol Oncol.

[143] Bisserier M, Wajapeyee N (2018). Mechanisms of resistance to EZH2 inhibitors in diffuse large B-cell lymphomas. Blood..

[144] Tap WD, Demetri G, Barnette P, Desai J, Kavan P, Tozer R (2012). Phase II study of ganitumab, a fully human anti-type-1 insulin-like growth factor receptor antibody, in patients with metastatic Ewing family tumors or desmoplastic small round cell tumors. J Clin Oncol.

[145] Van Cutsem E, Eng C, Nowara E, Swieboda-Sadlej A, Tebbutt NC, Mitchell E (2014). Randomized phase Ib/II trial of rilotumumab or ganitumab with panitumumabversus panitumumab alone in patients with wild-type KRAS metastatic colorectal cancer. Clin Cancer Res.

[146] Fuchs CS, Azevedo S, Okusaka T, Van Laethem J-L, Lipton LR, Riess H (2015). A phase 3 randomized, double-blind, placebo-controlled trial of ganitumab or placebo in combination with gemcitabine as first-line therapy for metastatic adenocarcinoma of the pancreas: the GAMMA trial. Ann Oncol.

[147] Juergens H, Daw NC, Geoerger B, Ferrari S, Villarroel M, Aerts I (2011). Preliminary efficacy of the anti-insulin-like growth factor type 1 receptor antibody figitumumab in patients with refractory Ewing sarcoma. J Clin Oncol.

[148] Olmos D, Postel-Vinay S, Molife LR, Okuno SH, Schuetze SM, Paccagnella ML (2010). Safety, pharmacokinetics, and preliminary activity of the anti-IGF-1R antibody figitumumab (CP-751,871) in patients with sarcoma and Ewing’s sarcoma: a phase 1 expansion cohort study. Lancet Oncol.

[149] Malempati S, Weigel B, Ingle AM, Ahern CH, Carroll JM, Roberts CT (2012). Phase I/II trial and pharmacokinetic study of cixutumumab in pediatric patients with refractory solid tumors and Ewing sarcoma: a report from the Children’s Oncology Group. J Clin Oncol.

[150] Schöffski P, Adkins D, Blay JY, Gil T, Elias AD, Rutkowski P (2013). An open-label, phase 2 study evaluating the efficacy and safety of the anti-IGF-1R antibody cixutumumab in patients with previously treated advanced or metastatic soft-tissue sarcoma or Ewing family of tumours. Eur J Cancer.

[151] Abdel-Wahab R, Varadhachary GR, Bhosale PR, Wang X, Fogelman DR, Shroff RT (2018). Randomized, phase I/II study of gemcitabine plus IGF-1R antagonist (MK-0646) versus gemcitabine plus erlotinib with and without MK-0646 for advanced pancreatic adenocarcinoma. J Hematol Oncol.

[152] Ramalingam SS, Spigel DR, Chen D, Steins MB, Engelman JA, Schneider CP (2011). Randomized phase II study of erlotinib in combination with placebo or R1507, a monoclonal antibody to insulin-like growth factor-1 receptor, for advanced-stage non-small-cell lung cancer. J Clin Oncol.

[153] Sclafani F, Kim TY, Cunningham D, Kim TW, Tabernero J, Schmoll HJ (2015). A randomized phase II/III study of dalotuzumab in combination with cetuximab and irinotecan in chemorefractory, KRAS wild-type, metastatic colorectal cancer. J Natl Cancer Inst.

[154] van Maldegem AM, Bovée JV, Peterse EF, Hogendoorn PC, Gelderblom H (2016). Ewing sarcoma: the clinical relevance of the insulin-like growth factor 1 and the poly-ADP-ribose-polymerase pathway. Eur J Cancer.

[155] Nanda R, Liu MC, Yau C, Shatsky R, Pusztai L, Wallace A, Chien AJ, et al. Effect of pembrolizumab plus neoadjuvant chemotherapy on pathologic complete response in women with early-stage breast cancer: an analysis of the ongoing phase 2 adaptively randomized I-SPY2 trial. JAMA Oncol. 2020:e196650. 10.1001/jamaoncol.2019.6650 PMC7058271 32053137

[156] Haluska P, Menefee M, Plimack ER, Rosenberg J, Northfelt D, LaVallee T (2014). Phase I dose-escalation study of MEDI-573, a bispecific, antiligand monoclonal antibody against IGFI and IGFII, in patients with advanced solid tumors. Clin Cancer Res.

[157] Iguchi H, Nishina T, Nogami N, Kozuki T, Yamagiwa Y, Yagawa K (2015). Phase I dose-escalation study evaluating safety, tolerability and pharmacokinetics of MEDI-573, a dual IGF-I/II neutralizing antibody, in Japanese patients with advanced solid tumours. Investig New Drugs.

[158] Fassnacht M, Berruti A, Baudin E, Demeure MJ, Gilbert J, Haak H, Kroiss M (2015). Linsitinib (OSI-906) versus placebo for patients with locally advanced or metastatic adrenocortical carcinoma: a double-blind, randomised, phase 3 study. Lancet Oncol.

[159] Chiappori AA, Otterson GA, Dowlati A, Traynor AM, Horn L, Owonikoko TK (2016). A randomized phase II study of linsitinib (OSI-906) versus topotecan in patients with relapsed small-cell lung cancer. Oncologist..

[160] Bendell JC, Jones SF, Hart L, Spigel DR, Lane CM, Earwood C (2015). A phase Ib study of linsitinib (OSI-906), a dual inhibitor of IGF-1R and IR tyrosine kinase, in combination with everolimus as treatment for patients with refractory metastatic colorectal cancer. Investig New Drugs.

[161] Barata P, Cooney M, Tyler A, Wright J, Dreicer R, Garcia JA (2018). A phase 2 study of OSI-906 (linsitinib, an insulin-like growth factor receptor-1 inhibitor) in patients with asymptomatic or mildly symptomatic (non-opioid requiring) metastatic castrate resistant prostate cancer (CRPC). Investig New Drugs.

[162] von Mehren M, George S, Heinrich MC, Schuetze SM, Yap JT, Yu JQ (2020). Linsitinib (OSI-906) for the treatment of adult and pediatric wild-type gastrointestinal stromal tumors, a SARC phase II study. Clin Cancer Res.

[163] Aiken R, Axelson M, Harmenberg J, Klockare M, Larsson O, Wassberg C (2017). Phase I clinical trial of AXL1717 for treatment of relapsed malignant astrocytomas: analysis of dose and response. Oncotarget..

[164] Holohan C, Van Schaeybroeck S, Longley DB, Johnston PG (2013). Cancer drug resistance: an evolving paradigm. Nat Rev Cancer.

[165] Buck E, Gokhale PC, Koujak S, Brown E, Eyzaguirre A, Tao N (2010). Compensatory insulin receptor (IR) activation on inhibition of insulin-like growth factor-1 receptor (IGF-1R): rationale for cotargeting IGF-1R and IR in cancer. Mol Cancer Ther.

[166] Rostoker R, Abelson S, Bitton-Worms K, Genkin I, Ben-Shmuel S, Dakwar M (2015). Highly specific role of the insulin receptor in breast cancer progression. Endocr Relat Cancer.

[167] Forest A, Amatulli M, Ludwig DL, Damoci CB, Wang Y, Burns CA (2015). Intrinsic resistance to cixutumumab is conferred by distinct isoforms of the insulin receptor. Mol Cancer Res.

[168] Ulanet DB, Ludwig DL, Kahn CR, Hanahan D (2010). Insulin receptor functionally enhances multistage tumor progression and conveys intrinsic resistance to IGF-1R targeted therapy. Proc Natl Acad Sci U S A.

[169] Garofalo C, Manara MC, Nicoletti G, Marino MT, Lollini PL, Astolfi A (2011). Efficacy of and resistance to anti-IGF-1R therapies in Ewing’s sarcoma is dependent on insulin receptor signaling. Oncogene..

[170] Seguin L, Desgrosellier JS, Weis SM, Cheresh DA (2015). Integrins and cancer: regulators of cancer stemness, metastasis, and drug resistance. Trends Cell Biol.

[171] Saegusa J, Yamaji S, Ieguchi K, Wu CY, Lam KS, Liu FT (2009). The direct binding of insulin-like growth factor-1 (IGF-1) to integrin alphavbeta3 is involved in IGF-1 signaling. J Biol Chem.

[172] Shin DH, Lee HJ, Min HY, Choi SP, Lee MS, Lee JW (2013). Combating resistance to anti-IGFR antibody by targeting the integrin β3-Src pathway. J Natl Cancer Inst.

[173] Wan X, Yeung C, Heske C, Mendoza A, Helman LJ (2015). IGF-1R inhibition activates a YES/SFK bypass resistance pathway: rational basis for co-targeting IGF-1R and Yes/SFK kinase in rhabdomyosarcoma. Neoplasia..

[174] Desbois-Mouthon C, Baron A, Blivet-Van Eggelpoël MJ, Fartoux L, Venot C, Bladt F (2009). Insulin-like growth factor-1 receptor inhibition induces a resistance mechanism via the epidermal growth factor receptor/HER3/AKT signaling pathway: rational basis for cotargeting insulin-like growth factor-1 receptor and epidermal growth factor receptor in hepatocellular carcinoma. Clin Cancer Res.

[175] Shin DH, Min HY, El-Naggar AK, Lippman SM, Glisson B, Lee HY (2011). Akt/mTOR counteract the antitumor activities of cixutumumab, an anti-insulin-like growth factor I receptor monoclonal antibody. Mol Cancer Ther.

[176] Knowlden JM, Gee JM, Barrow D, Robertson JF, Ellis IO, Nicholson RI (2011). erbB3 recruitment of insulin receptor substrate 1 modulates insulin-like growth factor receptor signalling in oestrogen receptor-positive breast cancer cell lines. Breast Cancer Res.

[177] Abraham J, Prajapati SI, Nishijo K, Schaffer BS, Taniguchi E, Kilcoyne A (2011). Evasion mechanisms to Igf1r inhibition in rhabdomyosarcoma. Mol Cancer Ther.

[178] Potratz JC, Saunders DN, Wai DH, Ng TL, McKinney SE, Carboni JM (2010). Synthetic lethality screens reveal RPS6 and MST1R as modifiers of insulin-like growth factor-1 receptor inhibitor activity in childhood sarcomas. Cancer Res.

[179] Huang F, Hurlburt W, Greer A, Reeves KA, Hillerman S, Chang H (2010). Differential mechanisms of acquired resistance to insulin-like growth factor-i receptor antibody therapy or to a small-molecule inhibitor, BMS-754807, in a human rhabdomyosarcoma model. Cancer Res.

[180] Heske CM, Yeung C, Mendoza A, Baumgart JT, Edessa LD, Wan X (2016). The role of PDGFR-β activation in acquired resistance to IGF-1R blockade in preclinical models of rhabdomyosarcoma. Transl Oncol.

[181] Gvozdenovic A, Boro A, Born W, Muff R, Fuchs B (2017). A bispecific antibody targeting IGF-IR and EGFR has tumor and metastasis suppressive activity in an orthotopic xenograft osteosarcoma mouse model. Am J Cancer Res.

[182] Zhao H, Desai V, Wang J, Epstein DM, Miglarese M, Buck E (2012). Epithelial-mesenchymal transition predicts sensitivity to the dual IGF-1R/IR inhibitor OSI-906 in hepatocellular carcinoma cell lines. Mol Cancer Ther.

[183] Nomura S (2019). Identification, friend or foe: vimentin and α-smooth muscle actin in cancer-associated fibroblasts. Ann Surg Oncol.

[184] Choi J, Gyamfi J, Jang H, Koo JS (2018). The role of tumor-associated macrophage in breast cancer biology. Histol Histopathol.

[185] Lin Y, Xu J, Lan H (2019). Tumor-associated macrophages in tumor metastasis: biological roles and clinical therapeutic applications. J Hematol Oncol.

[186] Ruffell B, Coussens LM (2015). Macrophages and therapeutic resistance in cancer. Cancer Cell.

[187] Ireland L, Santos A, Ahmed MS, Rainer C, Nielsen SR, Quaranta V (2016). Chemoresistance in pancreatic cancer is driven by stroma-derived insulin-like growth factors. Cancer Res.

[188] Tommelein J, De Vlieghere E, Verset L, Melsens E, Leenders J, Descamps B (2018). Radiotherapy-activated cancer-associated fibroblasts promote tumor progression through paracrine IGF1R activation. Cancer Res.

[189] Triplett TA, Cardenas KT, Lancaster JN, Hu Z, Selden HJ, Jasso GJ (2016). Endogenous dendritic cells from the tumor microenvironment support T-ALL growth via IGF1R activation. Proc Natl Acad Sci U S A.

[190] Lee JS, Kang JH, Boo HJ, Hwang SJ, Hong S, Lee SC (2015). STAT3-mediated IGF-2 secretion in the tumour microenvironment elicits innate resistance to anti-IGF-1R antibody. Nat Commun.

[191] Leonetti A, Sharma S, Minari R, Perego P, Giovannetti E, Tiseo M (2019). Resistance mechanisms to osimertinib in EGFR-mutated non-small cell lung cancer. Br J Cancer.

[192] Lee H, Kim N, Yoo YJ, Kim H, Jeong E, Choi S (2018). β-catenin/TCF activity regulates IGF-1R tyrosine kinase inhibitor sensitivity in colon cancer. Oncogene..

[193] Oh SC, Sohn BH, Cheong JH, Kim SB, Lee JE, Park KC (2018). Clinical and genomic landscape of gastric cancer with a mesenchymal phenotype. Nat Commun.

[194] Cohen-Sinai T, Cohen Z, Werner H, Berger R (2017). Identification of BRCA1 as a potential biomarker for insulin-like growth factor-1 receptor targeted therapy in breast cancer. Front Endocrinol (Lausanne).

[195] Trautmann M, Menzel J, Bertling C, Cyra M, Cyra M, Isfort I (2017). FUS-DDIT3 fusion protein-driven IGF-IR signaling is a therapeutic target in myxoid liposarcoma. Clin Cancer Res.

[196] Mancarella C, Casanova-Salas I, Calatrava A, Ventura S, Garofalo C, Rubio-Briones J (2015). ERG deregulation induces IGF-1R expression in prostate cancer cells and affects sensitivity to anti-IGF-1R agents. Oncotarget..

[197] Huang F, Chang H, Greer A, Hillerman S, Reeves KA, Hurlburt W (2015). IRS2 copy number gain, KRAS and BRAF mutation status as predictive biomarkers for response to the IGF-1R/IR inhibitor BMS-754807 in colorectal cancer cell lines. Mol Cancer Ther.

[198] Patel M, Gomez NC, McFadden AW, Moats-Staats BM, Wu S, Rojas A (2014). PTEN deficiency mediates a reciprocal response to IGFI and mTOR inhibition. Mol Cancer Res.

[199] Wang Q, Wei F, Lv G, Li C, Liu T, Hadjipanayis CG (2013). The association of TP53 mutations with the resistance of colorectal carcinoma to the insulin-like growth factor-1 receptor inhibitor picropodophyllin. BMC Cancer.

[200] Pavlicek A, Lira ME, Lee NV, Ching KA, Ye J, Cao J (2013). Molecular predictors of sensitivity to the insulin-like growth factor 1 receptor inhibitor Figitumumab (CP-751,871). Mol Cancer Ther.

[201] McCaffery I, Tudor Y, Deng H, Tang R, Suzuki S, Badola S (2013). Putative predictive biomarkers of survival in patients with metastatic pancreatic adenocarcinoma treated with gemcitabine and ganitumab, an IGF1R inhibitor. Clin Cancer Res.

[202] Lee HJ, Pham PC, Hyun SY, Baek B, Kim B, Kim Y (2018). Development of a 4-aminopyrazolo[3,4-d]pyrimidine-based dual IGF1R/Src inhibitor as a novel anticancer agent with minimal toxicity. Mol Cancer.

[203] Kuenzi BM, Remsing Rix LL, Stewart PA, Fang B, Kinose F, Bryant AT (2017). Polypharmacology-based ceritinib repurposing using integrated functional proteomics. Nat Chem Biol.

[204] Russo A, Paret C, Alt F, Burhenne J, Fresnais M, Wagner W (2019). Ceritinib-induced regression of an insulin-like growth factor-driven neuroepithelial brain tumor. Int J Mol Sci.

[205] Vewinger N, Huprich S, Seidmann L, Russo A, Alt F, Bender H (2019). IGF1R is a potential new therapeutic target for HGNET-BCOR brain tumor patients. Int J Mol Sci.

[206] Reuveni H, Flashner-Abramson E, Steiner L, Makedonski K, Song R, Shir A (2013). Therapeutic destruction of insulin receptor substrates for cancer treatment. Cancer Res.

[207] Ibuki N, Ghaffari M, Reuveni H, Pandey M, Fazli L, Azuma H (2014). The tyrphostin NT157 suppresses insulin receptor substrates and augments therapeutic response of prostate cancer. Mol Cancer Ther.

[208] Yang Y, Chan JY, Temiz NA, Yee D (2018). Insulin receptor substrate suppression by the tyrphostin NT157 inhibits responses to insulin-like growth factor-I and insulin in breast cancer cells. Horm Cancer.

[209] Janku F, Huang HJ, Angelo LS, Kurzrock R (2013). A kinase-independent biological activity for insulin growth factor-1 receptor (IGF-1R): implications for inhibition of the IGF-1R signal. Oncotarget..

[210] Pian L, Wen X, Kang L, Li Z, Nie Y, Du Z (2018). Targeting the IGF1R pathway in breast cancer using antisense lncRNA-mediated promoter cis competition. Mol Ther Nucleic Acids.

[211] Kumar AS, Rayala SK, Venkatraman G (2018). Targeting IGF1R pathway in cancer with microRNAs: How close are we?. RNA Biol.

